# Relational memory weakness in autism despite the use of a controlled encoding task

**DOI:** 10.3389/fpsyg.2023.1210259

**Published:** 2023-08-24

**Authors:** Greta N. Minor, Deborah E. Hannula, Andrew Gordon, J. Daniel Ragland, Ana-Maria Iosif, Marjorie Solomon

**Affiliations:** ^1^Department of Psychology, University of Wisconsin-Milwaukee, Milwaukee, WI, United States; ^2^Department of Psychiatry & Behavioral Sciences, University of California, Davis, Davis, CA, United States

**Keywords:** ASD, autism, episodic memory, relational memory, eye tracking

## Abstract

**Introduction:**

Recent work challenged past findings that documented relational memory impairments in autism. Previous studies often relied solely on explicit behavioral responses to assess relational memory integrity, but successful performance on behavioral tasks may rely on other cognitive abilities (e.g., executive functioning) that are impacted in some autistic individuals. Eye-tracking tasks do not require explicit behavioral responses, and, further, eye movements provide an indirect measure of memory. The current study examined whether memory-specific viewing patterns toward scenes differ between autistic and non-autistic individuals.

**Methods:**

Using a long-term memory paradigm that equated for complexity between item and relational memory tasks, participants studied a series of scenes. Following the initial study phase, scenes were re-presented, accompanied by an orienting question that directed participants to attend to either features of an item (i.e., in the item condition) or spatial relationships between items (i.e., in the relational condition) that might be subsequently modified during test. At test, participants viewed scenes that were unchanged (i.e., repeated from study), scenes that underwent an “item” modification (an exemplar switch) or a “relational” modification (a location switch), and scenes that had not been presented before. Eye movements were recorded throughout.

**Results:**

During study, there were no significant group differences in viewing directed to regions of scenes that might be manipulated at test, suggesting comparable processing of scene details during encoding. However, there was a group difference in explicit recognition accuracy for scenes that underwent a relational change. Marginal group differences in the expression of memory-based viewing effects during test for relational scenes were consistent with this behavioral outcome, particularly when analyses were limited to scenes recognized correctly with high confidence. Group differences were also evident in correlational analyses that examined the association between study phase viewing and recognition accuracy and between performance on the Picture Sequence Memory Test and recognition accuracy.

**Discussion:**

Together, our findings suggest differences in the integrity of relational memory representations and/or in the relationships between subcomponents of memory in autism.

## 1. Introduction

Autism is a neurodevelopmental disorder characterized by persistent difficulties with social interaction and communication, in addition to the presence of restricted and repetitive behaviors, interests, or activities (American Psychiatric Association, 2013). Although these behavioral hallmarks are criterial for an autism diagnosis, other aspects of cognition are also atypical in autism. For example, weaknesses in executive functioning and attention are reliably reported (Wainwright-Sharp and Bryson, 1993; Burack, 1994; Belmonte and Yurgelun-Todd, 2003; Landry and Bryson, 2004; Solomon et al., 2008, 2009; Mostert-Kerckhoffs et al., 2015; Lai et al., 2017; see Demetriou et al. (2018), Keehn et al. (2013) for reviews), and studies indicate that weaknesses in episodic memory are present as well (see Boucher et al. (2012), Cooper and Simons (2019), Desaunay et al. (2020a), Griffin et al. (2021) for reviews). Notably, differences in episodic memory have not always been reported in past work. One reason for contradictory findings may be that tasks used in some prior studies were susceptible to other forms of cognitive dysfunction in autistic individuals. For instance, direct tests of memory (e.g., recognition tasks that require deliberative processing and decision making) may be more reliant on executive functioning abilities than indirect measures of memory (e.g., measures that do not require explicit memory decisions), and executive functioning abilities are a well-documented weakness in autistic individuals. Additionally, some published studies used incidental encoding tasks (i.e., learning tasks without explicit instructions to memorize materials), which are likely to be more challenging for autistic individuals, who often show attentional difficulties relative to their non-autistic peers. Therefore, it is possible that previously reported memory difficulties in autism are a consequence of conflated cognitive requirements of specific tasks that have been used rather than evidence for true memory difficulties. The current study was designed to help adjudicate conflicting findings by employing both direct (i.e., explicit recognition) and indirect (i.e., eye-tracking) measures of memory performance in a task with experimental conditions matched for difficulty and more controlled encoding requirements.

Predicted episodic memory weaknesses in autistic persons are not unwarranted, as there are documented structural and functional connectivity differences in brain regions that contribute to episodic memory in autism. Research conducted with non-autistic participants reports that dissociable regions of the medial temporal lobes (MTL), including the hippocampus and perirhinal cortex, support long-term declarative memory (Eichenbaum et al., 2007; Konkel et al., 2008; Ranganath, 2010). Past studies indicate that the hippocampus is critical for the binding of associative, spatial, and temporal relationships between items in memory (i.e., relational memory), while the perirhinal cortex is identified as a key player in item-specific memory (Ryan et al., 2000; Davachi et al., 2003; Hannula et al., 2006; Staresina and Davachi, 2008; see Davachi (2006) for review). Consistent with behavioral reports of relational memory difficulties in autistic individuals, structural abnormalities in the hippocampus are reported in postmortem studies in this population (Bauman and Kemper, 2005; Fetit et al., 2021) and in structural imaging studies of hippocampal development (Reinhardt et al., 2020).

Neuroimaging studies, conducted with non-autistic individuals, also indicate that structures in the frontal and parietal lobes contribute to episodic memory encoding and retrieval (see Kim (2010), Spaniol et al. (2009) for reviews). The prefrontal cortex supports the organization of information in working memory during encoding, source monitoring during retrieval, post-retrieval selection of goal-relevant information, and self-referential processing that permits the integration of retrieved memories with prior knowledge (e.g., Dobbins and Wagner, 2005; Schlichting and Preston, 2015; see Blumenfeld and Ranganath (2007), Fletcher and Henson (2001) for reviews). Activation in the posterior parietal cortex is associated with the subjective experience of recollection, high confidence source memory judgments, attention to retrieved content, and the online representation and maintenance of retrieved representations over time (Cabeza et al., 2008; Ciaramelli et al., 2017; see Moscovitch et al. (2016), Rugg and Vilberg (2013) for reviews).

Postmortem studies and structural imaging work indicate volumetric differences in frontal and parietal brain regions in autistic individuals relative to controls (Ecker et al., 2010; Fetit et al., 2021; although see Trontel et al., 2015), and functional neuroimaging studies demonstrate abnormalities in functional connectivity between the prefrontal cortex, parietal regions, and the hippocampus in autistic individuals (e.g., Ben Shalom, 2003; Barnea-Goraly et al., 2014; Cooper et al., 2017b; Banker et al., 2021; Li et al., 2022). For example, attenuated functional connectivity between the hippocampus and fronto-parietal networks is reported during retrieval, accompanied by lower levels of retrieval accuracy, in autistic individuals (Cooper et al., 2017b). Another study documents reduced activation in the left posterior hippocampus and enhanced PFC activation during encoding, which may indicate more effortful encoding for these individuals (Gaigg et al., 2015).

The combination of structural and functional differences in memory-associated brain areas observed in autism align with reported weaknesses on long-term memory tasks requiring retrieval of details diagnostic of the encoding experience (Boucher and Warrington, 1976; Boucher, 1981; Bowler et al., 1997). Specifically, autistic individuals make fewer subjective, recollection-related responses (e.g., “remember” responses in remember-know paradigms; e.g., Bowler et al., 2007; Cooper et al., 2015), exhibit reduced confidence in judgments of mnemonic accuracy (i.e., metamemory; e.g., Wojcik et al., 2013; Grainger et al., 2014; Cooper et al., 2016), and demonstrate poorer recall of autobiographical memories (e.g., Lind and Bowler, 2010). Consistent with these observations, individuals diagnosed with autism have a disproportionate weakness in relational memory with relatively intact memory for individual items (Bigham et al., 2010; Bowler et al., 2014; Desaunay et al., 2020b). Indeed, relational memory difficulties are documented across a range of stimuli (e.g., abstract and realistic objects, words, etc.) and across different types of relational memory tasks (e.g., inter-object, object-location, object-color, object-action, and object-voice pairing tasks; Lind and Bowler, 2009; Bigham et al., 2010; Bowler et al., 2014; Cooper et al., 2017b; Desaunay et al., 2020b). Such findings are in line with the *relational binding account* of episodic memory in autism (Bowler et al., 2011), which posits that autistic individuals show a selective weakness in hippocampus-dependent binding of items and contexts but a relative sparing of memory for items alone.

Importantly, the relational binding account has not always been supported by previous findings. Some studies report that autistic individuals show difficulties restricted to item memory (Solomon et al., 2016; Cooper et al., 2017a), weaknesses in both item and relational memory (Cooper et al., 2015; Massand and Bowler, 2015; Ring et al., 2016; Semino et al., 2018; Mogensen et al., 2020), or intact item and relational memory (Souchay et al., 2013; Lind et al., 2014; Ring et al., 2015, 2017; Hogeveen et al., 2020). One possible explanation for discrepant findings is that task complexity differed across item-specific and relational memory tasks in these experiments (e.g., as in Bowler et al., 2014). Indeed, in past work, tests of item-specific memory have typically required participants to recognize a single item from the encoding phase, while tests of relational memory required participants to remember multiple elements of the encoding scenario. Further supporting the potential influence of this confound on prior work, autistic individuals have shown difficulties with processing “complex” information (e.g., complex conceptual structure/organization of material and/or retrieval tasks that require higher levels of cognitive control) across a range of cognitive tasks (*complex information processing model*; Minshew and Goldstein, 1998, 2001). Thus, it is conceivable that reports from previous studies are in conflict because task demands are typically quite different for item-specific and relational memory tests.

To address this problem, Cooper et al. (2015) utilized a long-term memory task with item-specific and relational memory conditions that were well-matched for task difficulty. During encoding, autistic and non-autistic adults studied computer-generated scenes that contained pre-defined “critical” items. Subsequently, in a corresponding test phase, participants were presented with previously studied and new (i.e., never presented) scenes, and some of the studied scenes were modified. When scenes were modified, rather than repeated, the critical item was either replaced with a different exemplar (i.e., item-specific change) or had moved to a new spatial location (i.e., relational change). Participants were instructed to determine, for each test scene, whether it was repeated, modified, or new. Importantly, the experiment was designed so that memory for item-specific detail and spatial relationships was assessed in the context of the same set of scenes, and pilot testing had confirmed that performance was well-matched across conditions (Hannula et al., 2015). Results indicated that autistic individuals identified significantly fewer modified scenes in both the item-specific and relational memory conditions relative to their non-autistic peers and that autistic participants were less likely to endorse successfully identified scenes as recollected. Thus, when task-difficulty is well-matched across conditions, it appears that the memory weakness is not limited to relational memory (Cooper et al., 2015).

It is important to note, however, that much of the past work investigating long-term episodic memory in autistic individuals, including Cooper et al.’s (2015) study, has relied solely on explicit behavioral responses (e.g., button-press recognition responses). This is problematic because complex instructions and/or button-press mappings in these experiments depend on the integrity of additional cognitive processes (Luck and Gold, 2008) that are impacted in autistic individuals (e.g., cognitive control; Schmitt et al., 2018; see Tonizzi et al. (2021) for review). Moreover, other aspects of previously published studies (e.g., relatively uncontrolled encoding conditions) make it difficult to determine whether results provide evidence of true memory difficulties or are a secondary consequence of attentional and executive processing differences during encoding. For example, in Cooper et al.’s (2015) work, participants were instructed to try and remember the appearance and location of the objects in the scene. However, autistic individuals show difficulties with the disengagement of attention (see Keehn et al. (2013) for review) and inefficient attentional filtering of information (e.g., Burack, 1994; Murphy et al., 2014; Keehn et al., 2019), which may have interfered with the initial exploration and encoding of information in scenes during the study phase and may have led to reported memory weaknesses. In sum, specific task requirements may result in the conflation of cognitive processes that are differentially impacted in autistic individuals, and these differences may account for reported discrepancies in autistic performances on episodic memory tests.

Therefore, other methods may be useful in disentangling contradictory findings. One method used to index memory indirectly is eye tracking. An advantage of this method is that eye movements can be recorded throughout an experiment, which means that researchers can pinpoint when (i.e., at what stage of processing – encoding vs. retrieval) there are differences in performance (e.g., differences in scene exploration) that may contribute to reported memory difficulties in special populations. Past eye-tracking studies with healthy, college-age participants demonstrate that when a stimulus is presented repeatedly, participants make fewer fixations and sample fewer distinct regions of a picture with each repetition (i.e., Althoff and Cohen, 1999; Ryan et al., 2000, 2007; Heisz and Shore, 2008). Additionally, the number of fixations made during encoding is positively correlated with recognition accuracy during test (Pertzov et al., 2009; Molitor et al., 2014; Olsen et al., 2014) and, during retrieval, viewing patterns distinguish previously studied scenes that have been modified from those that are repeated without a change (e.g., Ryan et al., 2000).

In one representative example, Hannula et al. (2010a,b) used the task subsequently adopted by Cooper et al. (2015) but also incorporated a second, controlled encoding phase. During this second encoding phase, participants viewed the same set of scenes that were presented during the first encoding phase, but now each scene was accompanied by an orally-presented “yes/no” question orienting a participant’s attention to either the features of a “critical” item (i.e., an ‘item-specific’ orienting question) or to the spatial location of a “critical” item (i.e., a ‘spatial relational’ orienting question) that might be modified in the test phase. Use of orienting questions during the encoding task ensured that participants attended to the very same information that might be manipulated subsequently, meaning that any differences in retrieval performance were less likely due to differences in attention to critical objects during encoding. At test, participants spent more time fixating the critical regions of repeated (versus novel) scenes because attention had been directed to these regions by the orienting questions during the second encoding phase. Additionally, a disproportionate amount of time was spent viewing critical regions of modified (versus repeated) scenes, including the *empty* regions of scenes when a relational change had been made (i.e., the location originally occupied by the critical object, now empty). Because eye movements are more likely to be made toward objects than to empty regions of a scene (Yarbus, 1967), these viewing time differences represent particularly compelling evidence for the influence of relational memory on eye-movement behavior (see also Ryan et al. (2000)).

Further evidence for the sensitivity of eye movements to item-specific and relational memory comes from previous work with clinical populations. For instance, in the study described above (Hannula et al., 2010b), individuals diagnosed with schizophrenia showed a disproportionate deficit in the eye-movement-based relational memory effect relative to healthy comparison participants. This outcome is similar to impairments reported when amnesic patients with MTL damage are tested in comparable experiments. Specifically, amnesic patients show standard effects of stimulus repetition in patterns of viewing, but eye-movement-based relational memory effects are impaired (e.g., Ryan et al., 2000). In studies of autism, eye tracking has been used to examine the exploration of social stimuli (with differences in viewing reported; see Chita-Tegmark (2016); Papagiannopoulou et al. (2014) for reviews), but only a handful of previous studies have used this method to address questions about the integrity of long-term memory (Loth et al., 2011; Ring et al., 2017; Cooper et al., 2017a).

In general, published eye-tracking studies indicate that viewing effects (e.g., gaze time, number of fixations, fixation duration) are similar between autistic and non-autistic individuals during encoding, suggesting attention to scenes during encoding is unaffected in autism (Loth et al., 2011; Cooper et al., 2017a). However, when correlational analyses are conducted to examine associations between viewing patterns and subsequent memory, results suggest that viewing patterns may not predict subsequent memory performance to the same degree in autistic and non-autistic participants (Loth et al., 2011; Ring et al., 2017; Cooper et al., 2017a). It is proposed that these differences point to a problem at the time of retrieval, rather than encoding, in autistic individuals (Cooper et al., 2017a), since differences in memory performance occur during the retrieval phase and are accompanied by similar eye-movement patterns during encoding. Consistent with this conclusion, past work measuring retrieval-related viewing patterns indicate that fixation ‘reinstatement’ (i.e., extent to which viewing patterns from study are reinstated during test) is reduced for recollected scenes in autistic relative to non-autistic participants, while reinstatement patterns for non-recollected scenes are not different between groups (Cooper et al., 2017a), potentially indicating that memory weaknesses reported in autism are due to a disrupted recollection-related retrieval process (cf. Griffin et al., 2021).

To our knowledge, one eye-tracking study has explicitly used a relational memory task in an autism population (Ring et al., 2017), and results revealed between-groups differences in retrieval-related eye movements. During test phase trials, three locations were marked in previously studied scenes – one corresponding to the location that was occupied by a studied object and two previously unoccupied locations. On every trial, participants were either presented with the originally encoded object or a new, unstudied object. In each case, they were required to place the object in one of the marked scene locations. For “include” trials, they were to put the object in its originally studied location; for “exclude” trials, they were to put the object in one of the two new locations (i.e., process dissociation procedure; Jacoby, 1991). If unable to remember the object or the location, participants were told to choose one of the available locations (i.e., a measure of potential position-based bias for the set of counterbalanced new objects). Results indicated that both groups of participants were equally likely to place the object in its original location on “exclude” trials (a measure of implicit memory) but that individuals with autism were less likely to put the object in its original location on “include” trials (a measure of explicit memory). Eye-tracking results revealed that, during encoding, non-autistic individuals spent more time viewing objects that were subsequently placed correctly during test relative to autistic individuals. In addition, autistic participants spent less time looking at target locations during “include” trials and non-target locations during “exclude” trials compared to the non-autistic participants. Collectively, these results are consistent with reports that relational memory is disrupted in autism, and, further, differences were evident not only in direct measures of performance but also when memory was measured indirectly, using eye movement data.

In a key departure from previously published studies, eye-tracking data was recorded here in a task that examined both item-specific and relational memory. Importantly, as indicated earlier, a norming experiment demonstrated that these experimental conditions were equated for difficulty (Hannula et al., 2010b) to ensure viewing effects could not be attributed to differential task complexity. Specifically, we examined whether memory-specific viewing patterns to realistic, non-social scenes differed between autistic and non-autistic individuals. Participants first viewed a set of scenes while being instructed to memorize the scene. Following the initial study phase, scenes were re-presented, accompanied by an orienting question (e.g., “Is the hat on the chair?”). Participants were told to respond to the question, which encouraged them to attend to specific objects in the scenes that might be subsequently manipulated (i.e., exchanged with different exemplar or moved to different spatial location) during the test phase. This ‘orienting’ question was intended to reduce the burden on attentional resources and executive functions that may be compromised in autism. During test, participants viewed scenes that were unchanged (i.e., repeated from study), scenes that underwent an “item” change (an exemplar switch) or a “relational” change (a location switch), and scenes that were not presented during the encoding phase. Both direct (i.e., recognition responses) and indirect (i.e., eye movement) measures of memory were recorded.

Consistent with results reported in Cooper et al.’s (2015) study that used the same scenes and a similar task, one possibility was reduced explicit recognition accuracy for modified scenes in the autistic group, whether the change was item-specific or relational. The few studies examining eye-movement behavior in autism suggest that between-group differences in basic viewing patterns might not be evident during encoding. It is possible though that there may be reductions in the positive correlations between encoding-related eye movements and subsequent memory performance, as reported previously in autism (Loth et al., 2011; Ring et al., 2017; Cooper et al., 2017a). During test, eye movement effects sensitive to memory for spatial relationships might be selectively reduced in autism, an outcome consistent with the relational binding hypothesis (Bowler et al., 2011). However, if the problem in autism is related to the initial processing of relational information (e.g., during encoding), then use of an orienting question during the second study block should reduce or eliminate the relational memory difficulty because these questions encourage participants to attend to and process the same relationships that might be modified at test. In sum, use of direct and indirect measures of memory, together with well-matched item-specific and relational memory conditions, was expected to aid in disambiguating contradictory findings reported in the autism episodic memory literature.

## 2. Method

### 2.1. Participants

Forty participants (18 autistic, 22 non-autistic) were recruited during the second wave of data collection from a cohort-sequential study (*Neurodevelopment of cognitive control in autism: adolescence to young adulthood*; 1R01MH106518) of autistic and non-autistic persons without intellectual disability (IQ ≥ 70) through the University of California (UC) Davis MIND Institute and Imaging Research Center. Two participants in the non-autistic group were removed from analysis because the number of test block trials with unreliable eye-tracking data was more than two standard deviations above the group mean. Therefore, the sample carried forward for analysis included 18 autistic individuals and 20 non-autistic individuals. This sample size was comparable to, or greater than, the sample size from previously published studies using the same task (i.e., Cooper et al. (2015) – 24 participants per group; Hannula et al. (2010b) – 16 participants per group). With this sample size, we had sufficient power (80.4%) to detect large effects for group differences (*d* = 0.9) with alpha set to 0.05, two-tailed.

Written, informed consent was obtained from participants in accordance with the UC Davis Institutional Review Board. Participants received a gift card for their participation. To be included in the study, all participants were required to be between the ages of 12 and 24 and to have a Full Scale IQ of 70 or above on the Wechsler Abbreviated Scale of Intelligence – 2nd Edition (WASI-II; Wechsler, 2011). Participants were not permitted to be taking psychotropic medications at the time of their enrollment in the study. Participants were also excluded from participation if they had a diagnosis of epilepsy or another neurological disorder and/or if imaging was contraindicated. Autistic participants were required to have a community diagnosis of autism and were required to meet criteria for autism on a DSM-5 Criteria Checklist for autism (American Psychiatric Association, 2013) and on the Autism Diagnostic Observation Schedule – 2nd Edition (ADOS-2; Lord et al., 2000), which were administered by a licensed clinician at the UC Davis MIND Institute. Non-autistic participants were not included in the study if they had a community diagnosis of autism, attention-deficit/hyperactivity disorder, or any neurodevelopmental disorder, had a first-degree family member with autism, had reported Axis I psychopathology, or surpassed a cut-off value of 11 on the Social Communication Questionnaire (SCQ; Rutter et al., 2003), suggestive of an autism diagnosis.

Table 1 provides basic descriptive statistics for each group on the following characteristics: gender, chronological age, WASI-II (Wechsler, 2011) Full Scale IQ (FSIQ-4), and WASI-II index scores (Verbal Comprehension Index [VCI] and Perceptual Reasoning Index [PRI]). There were no significant differences between groups on age, WASI-II FSIQ-4, or WASI-II index scores, *F*’s ≤ 1.61, *p’*s ≥ 0.21. In Table 1, scores on the semi-structured ADOS-2 (Lord et al., 2000) are also provided for individuals in the autistic group, including the calibrated severity score (CSS) and severity scores in the Social Affect (SA) and Restricted, Repetitive Behavior (RRB) domains. Table 2 presents scores on select tests from the NIH Toolbox® Cognition Battery used to assess symptoms related to inattention/impulsivity, executive dysfunction, working memory, and episodic memory (Akshoomoff et al., 2013), including scores on the Flanker Inhibitory Control and Attention Test (FICA), Dimensional Change Card Sort Test (DCCS), Picture Sequence Memory Test (PSM), and List Sorting Working Memory Test (LSWM). There were significant differences between groups on two executive functioning tasks (FICA, DCCS), Welch’s *F’s* ≥ 6.20, *p*’s ≤ 0.020, *ω^2^*’s ≥ 0.12, and on an episodic memory test (PSM), *F* (1, 36) = 5.35, *p* = 0.027, *η_p_^2^* = 0.13, with higher scores in the non-autistic group compared to the autistic group across all three measures. There were no significant group differences on a working memory task (LSWM), *F* (1, 36) = 0.52, *p* = 0.47.

**Table 1:** Demographic and clinical characteristics of the sample.

	Autistic (*n* = 18)	Non-Autistic (*n* = 20)
Female	7 (39%)	5 (25%)
Male	11 (61%)	15 (85%)
Age	20.68 (2.71; 16.42–24.83)	21.28 (2.39; 17.08–24.92)
FSIQ-4	103.11 (12.22; 76–125)	108.60 (14.20; 79–129)
VCI	102.61 (10.42; 85–120)	105.5 (15.81; 73–137)
PRI	103.11 (16.57; 68–131)	109.35 (14.76; 83–140)
ADOS CSS	7.06 (2.10; 4–10)	–
ADOS SA Severity	7.33 (2.00; 3–10)	–
ADOS RRB Severity	6.5 (2.94; 1–10)	–

Data are reported as frequencies (percentages) for categorical variables and means (standard deviations; ranges) for continuous variables.

FSIQ-4 (Full Scale IQ composed of 4 indices); VCI (Verbal Comprehension Index); PRI (Perceptual Reasoning Index); ADOS (Autism Diagnostic Observation Schedule); ADOS CSS (ADOS Calibrated Severity Score); ADOS SA Severity (ADOS Social Affect Severity); ADOS RRB Severity (ADOS Restricted, Repetitive Behavior Severity).

**Table 2:** NIH toolbox^®^ cognition battery scores for autistic and non-autistic participants.

	Autistic (*n* = 18)	Non-autistic (*n* = 20)
Flanker Inhibitory Control and Attention Test (FICA)	103.89 (8.17; 90–114)	111.45 (4.26; 104–117)
Dimensional Change Card Sort Test (DCCS)	105.11 (12.12; 81–120)	113 (6.14; 101–120)
Picture Sequence Memory Test (PSM)	107.17 (11.25; 86–123)	117 (14.54; 95–136)
List Sorting Working Memory Test (LSWM)	110.06 (12.29; 90–136)	112.55 (8.85; 97–128)

Data are reported as means (standard deviations; ranges).

### 2.2. Materials and apparatus

**Figure 1:**
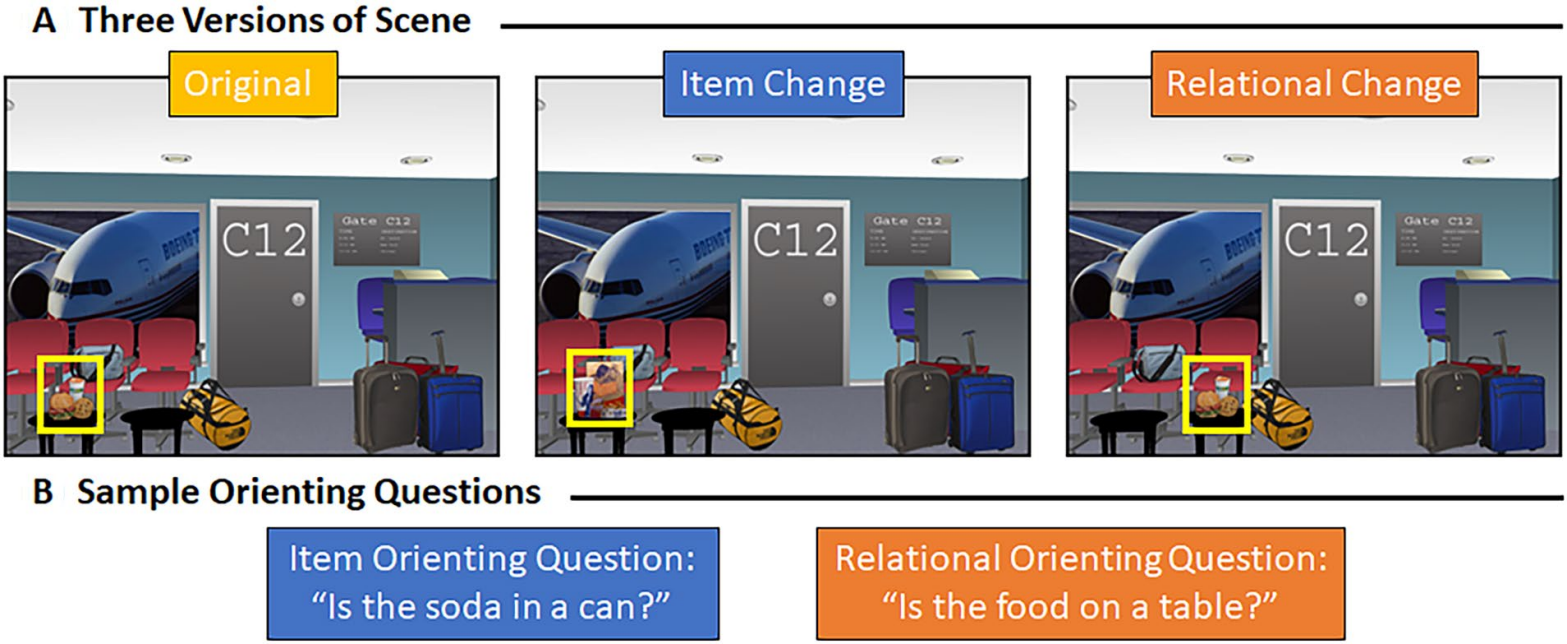
Representative scene and associated orienting questions. **(A)** Example of a representative scene – the original scene, the version of that scene with an item manipulation, and the version of that scene with a relational manipulation. **(B)** Item (in blue) and relational (in orange) orienting question for the scene shown above **(A)**.

Sixty-four computer-generated indoor and outdoor scenes (800 × 600 pixels) created using Punch! Home Design Software (Encore, Inc., El Segundo, CA) by Hannula et al. (2006, 2010b) were used in the current study. Three versions of each scene were developed – an original version, a version in which a designated critical item was switched with a different exemplar (i.e., an item manipulation), and a version in which that same critical item had been moved to a similarly plausible location (i.e., a relational manipulation; see Figure 1A). The total stimulus sample included 192 scenes. When critical objects switched spatial locations in the relational condition, objects were moved equally often from left, in the original scene, to right, in the manipulated scene, and vice versa. Scenes were presented at a resolution of 1,012 × 762 pixels, and scenes subtended 28.61 (width) by 21.74 (height) degrees of visual angle, from a viewing distance of 70 cm. Scenes were displayed on a monitor with 1,980 × 1,200-pixel resolution and a refresh rate of 60 Hz. Additionally, two orienting questions were created for each scene. One question was designed to orient attention to the features of a critical object and the other to the spatial relationship between a critical object and its surroundings (examples are provided in Figure 1B). The purpose of the orienting question was to direct the viewer’s attention to critical properties of the scenes that might be manipulated during the subsequent test block.

Eye movements were recorded with an Eyelink 1,000 Plus eye-tracking system (SR Research LTD: Ontario, Canada). This system has a temporal resolution of 1,000 Hz and head-supported spatial resolution of 0.01°. Eye movements were identified as saccades using an automated algorithm that requires a minimum velocity of 30°/s and a minimum acceleration of 8,000°/s^2^. Experiment Builder software package (SR Research LTD: Ontario, Canada) was used to display the experiment, and Data Viewer software package (SR Research LTD: Ontario, Canada) was used to extract the eye-tracking data.

### 2.3. Design and procedure

After participants gave their consent to participate, they were seated 70 cm from the computer monitor and a chinrest was adjusted to a comfortable position. An automated 9-point calibration process was then performed to align fixations with screen coordinates before the experiment began; this process was repeated as necessary until calibration was successful, and a drift correction procedure was used before each trial to ensure accurate tracking throughout the experiment. Prior to completing the experiment, instructions were provided. Twelve practice study trials (six each in Study Blocks 1 and 2) and eight practice test trials were used to ensure that participants understood the task. During the practice test trials, participants were given feedback on their performance. Scenes viewed during study and test were presented side-by-side to afford participants the opportunity to become familiar with the types of scene manipulations they may encounter. Eye movements were recorded in each phase of the experiment.

#### 2.3.1. Study Block 1

**Figure 2:**
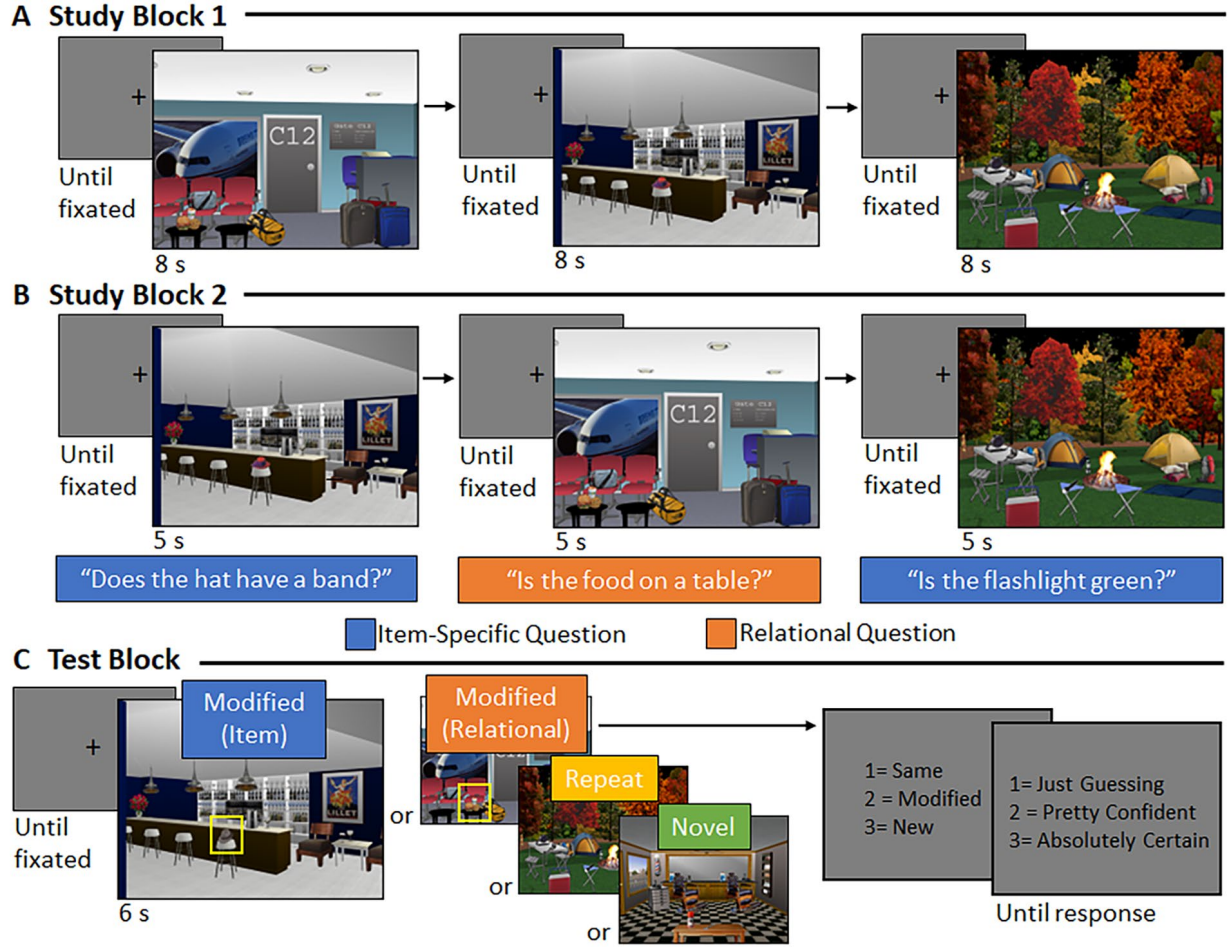
Trial structure and event timing. **(A)** During Study Block 1, central fixation was followed by a scene (8 s). **(B)** During Study Block 2, central fixation was followed by a scene (5 s), accompanied by an orally presented orienting question to which participants responded via button press. **(C)** During the test block, central fixation was followed by a scene (6 s). Participants indicated via button press whether the scene was the “same,” “modified,” or “new” and provided a confidence rating, when prompted.

Following practice, participants were shown 48 scenes during Study Block 1 (see Figure 2A). Sixteen of these scenes were ‘repeated’ during test (i.e., same version of the scene was re-presented), 16 underwent an item manipulation at test (i.e., henceforth referred to as the “item” condition), and 16 underwent a relational manipulation at test (i.e., henceforth referred to as the “relational” condition). Participants were instructed to view the scenes and attempt to commit each scene to memory. Every trial began with a central fixation cross; the trial could not be initiated by the experimenter until the participant fixated the center of the screen. Each scene was presented for a duration of 8 s.

#### 2.3.2. Study Block 2

During Study Block 2, the same 48 scenes were presented again in a new random order (see Figure 2B). When participants fixated the center of the screen, the experimenter initiated the trial, and a scene was presented for 5 s. Now, each scene was accompanied by a corresponding orienting question (pre-recorded and presented over speakers), initiated 500 ms after scene onset. The question directed the participant’s attention either to features of a critical object (if the scene was assigned to the “item” condition) or to the spatial relationship between a critical object and its surroundings (if the scene was assigned to the “relational” condition). For scenes assigned to the “repeated” condition, half were presented with an item-specific orienting question and half were presented with a relational orienting question. Participants were instructed to respond “yes,” “no,” or “don’t know” to the orienting question via a button press, while the picture was in view.

#### 2.3.3. Test Block

**Figure 3:**
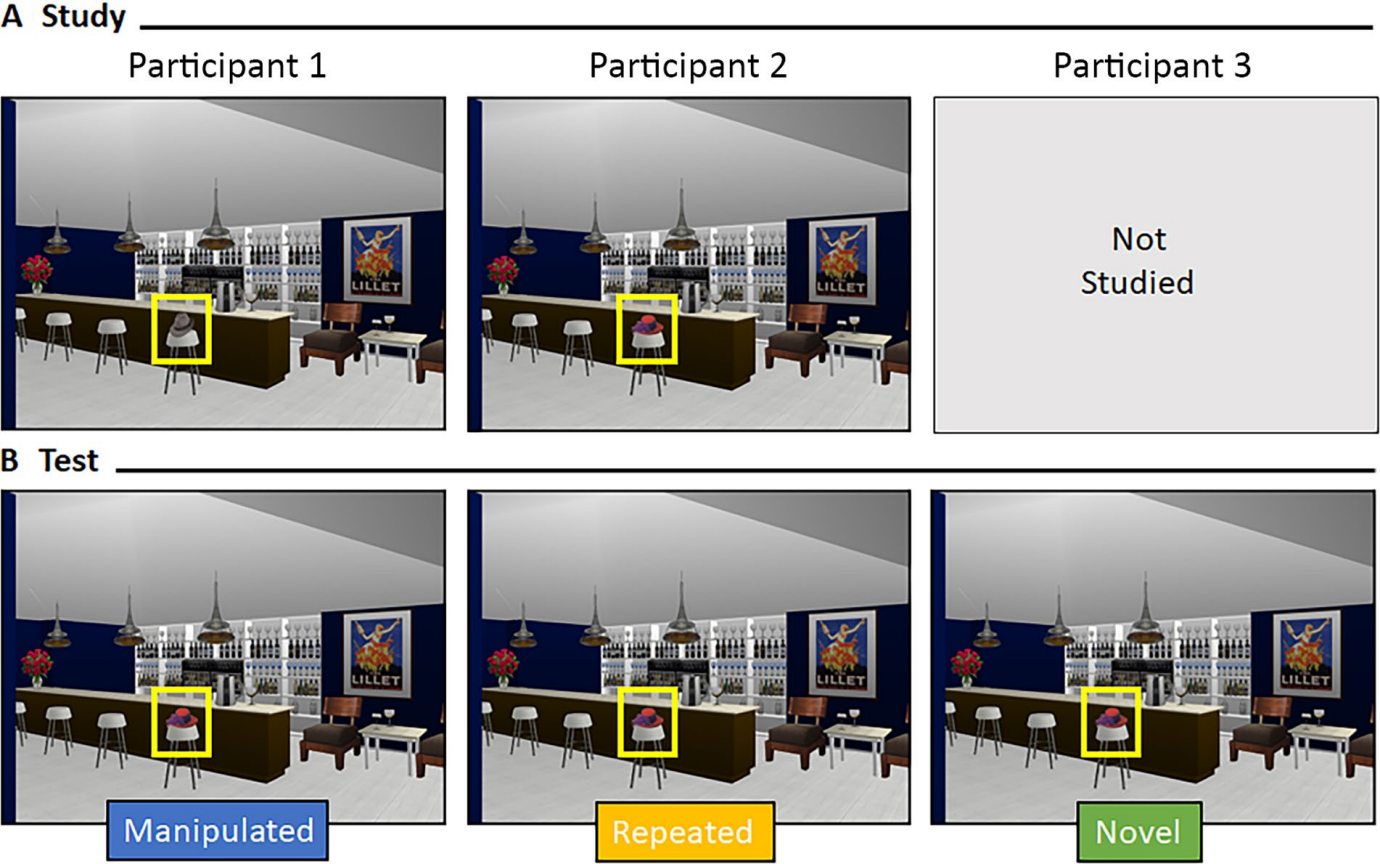
Yoking procedure at test. Representative example of a yoked scene during **(A)** study and **(B)** test for three different participants. Scenes during test were identical across participants but varied by encoding history.

Participants saw 64 scenes during the Test Block (see Figure 2C). Sixteen scenes were the exact image seen during study (i.e., “repeated” scenes), 16 scenes had undergone an item manipulation (i.e., “item” scenes), 16 scenes had undergone a relational manipulation (i.e., “relational” scenes), and 16 scenes were new (i.e., “novel” scenes). Critically, a yoked design was used; three participants saw the exact same version of a scene during test, but different encoding experiences meant the scene was manipulated for one participant, repeated for another, and novel for a third (see Figure 3). This yoked design means that any differences in viewing, across conditions, could not be due to differences in features of the scenes presented during the test phase. Instead, any differences in viewing patterns would be directly attributable to differences in encoding history. Scenes were presented equally often as repeated, manipulated, and novel across participants.

Following central fixation, the experimenter initiated the trial, and a scene was presented for 6 s. After the scene disappeared from the screen, participants were prompted to respond via button press whether the scene was the “same” as one they had studied, had been “modified” somehow, or was “new.” Then, participants were asked to rate their recognition confidence on a scale from 1 (“just guessing”) to 3 (“absolutely certain”) with a button-press response. In each case, response options remained on the screen until a response was made. At the end of the experiment, participants were debriefed.

#### 2.3.4. Data processing and analysis

Trials were flagged and removed from analyses when eye position was lost or unreliable. As in previously published work (e.g., Hannula et al., 2010b), trials were removed if the total viewing time directed to the scene was less than 65% of the trial duration. This resulted in the loss of 2.14% of trials (*SD* = 2.98%) across autistic and non-autistic participants. Two participants from the non-autistic group were removed from all analyses because the number of test block trials flagged as bad was more than two standard deviations above the group mean (28 and 55% of the trials, respectively). To examine differences in processing of and attention toward critical items, orienting question accuracy was calculated for button-press responses made during Study Block 2. Corrected recognition scores were calculated to determine whether explicit memory performance during the Test Block differed between groups. As was done by Cooper et al. (2015), the percentage of studied (repeated and modified) scenes mistakenly endorsed as “new” (i.e., Novel False Alarms) was subtracted from the percentage of novel scenes that were identified correctly (i.e., Novel Hits) to examine *memory for scenes*. Corrected recognition scores sensitive to *memory for scene detail* were calculated separately for the item and relational conditions by subtracting the percentage of repeated scenes incorrectly endorsed as “modified” from the percentage of item and relational scenes identified correctly, respectively. This measure provides us with information about how effectively participants could discriminate between studied scenes that went on to be manipulated and studied scenes that remained the same.

Three regions of interest were drawn for each scene to examine viewing effects. One of these regions marked the boundaries of the whole scene (i.e., “scene” region), one marked the current location of the critical object (i.e., “filled” region), and one marked the location where the critical object used to be located (i.e., “now-empty” region) when a relational change was made. The boundaries of the “filled” and “empty” regions were drawn in Adobe Photoshop to extend 25 pixels beyond the horizontal and vertical limits of the critical object. Fixations outside the bounds of the “scene” region were discarded from analyses, and total viewing time, used as the denominator in our proportion of total viewing time measures, was the summed duration of fixations made to the scene itself (rather than the full duration of scene presentation; see Hannula et al. (2010a) for details). For the Study Blocks, regions of interest analyses were based on viewing directed to each scene’s “filled” location (i.e., location occupied by the critical object). Scenes presented during the study blocks (i.e., scenes in the repeated, item, and relational conditions) were subdivided based on whether they were presented with an item-specific or relational orienting question during Study Block 2. For the Test Block, region of interest analyses were based on viewing directed to the “filled” location for scenes that underwent an *item change* (along with their yoked repeated and novel counterparts) and viewing directed to both the “filled” and “empty” locations for scenes that underwent a *relational change* (along with their yoked repeated and novel counterparts).

To determine whether there were differences in viewing between groups during Study Blocks 1 and 2, the average number of fixations made to whole scenes (collapsed across conditions) was examined, along with the proportion of total viewing time directed to the filled critical region of scenes accompanied by item-specific and relational orienting questions (collapsed across to-be-repeated and to-be-manipulated scenes). As in previous work (Hannula et al., 2010b), we calculated two separate memory indices to examine viewing patterns from the Test Block. Our first index, *memory for repetition*, was used to determine whether there were differences in viewing due to memory for the scenes themselves, absent any modification. Viewing of the critical region(s) within novel scenes (i.e., scenes presented for the first time during test) was subtracted from viewing of the analogous region(s) within repeated scenes (presented during study and test). The second index, *memory for detail*, was used to determine whether item-specific and/or relational changes affected viewing of the critical region(s). In this case, viewing of the critical region(s) within repeated scenes (presented during study and test) was subtracted from viewing of the analogous region(s) within manipulated scenes (in which an item or relational change occurred at test). We utilized these two calculated indices to examine two eye-movement measures: the proportion of total viewing time directed to critical scene region(s) and the duration of the first gaze (in ms) to the filled region. For the first gaze analysis, the durations of consecutive fixations to the filled region in the first gaze following scene presentation were summed. The first gaze began with the first entry into the filled region and ended when the participant looked at a different scene location. Empty locations, in the relational condition, were not included in the first gaze analysis because so few fixations were made to that part of the scene.

##### 2.3.4.1. Statistical contrasts

Analyses were conducted in SPSS Statistics (Version 28.0). All tests were two-sided and *p*-values <0.05 were considered statistically significant. Levene’s test was used to examine homogeneity of variances before conducting independent samples *t*-tests and repeated measures ANOVAs. Age and gender were included as covariates when marginal or significant group differences were documented. Partial eta-squared (*η_p_^2^*) and Cohen’s *d* were calculated as effect size indices.

Additionally, Bayes factors, giving evidence for the null hypothesis over the alternative hypothesis (*BF_01_*), were calculated to determine whether reported results were likely to have been obtained under the null or alternative hypothesis or whether results did not favor either hypothesis. A Bayes factor *(BF_01_)* greater than 3 provides evidence for the null hypothesis, and a value less than 0.33 provides evidence for the alternative hypothesis, while any value between 3 and 0.33 is inconclusive.

Finally, Pearson’s correlations were calculated to examine associations between viewing patterns during study (i.e., proportion of total viewing time to filled regions in studied scenes), viewing patterns during test (i.e., detailed-based proportion of total viewing time and first gaze duration), and recognition memory performance (i.e., corrected recognition scores). Specifically, we examined four types of associations: 1) association between critical region viewing for scenes paired with *item* orienting questions during study and detail-based viewing for scenes with an *item* change during test, 2) association between critical region viewing for scenes paired with *relational* orienting questions during study and detail-based viewing for scenes with a *relational* condition during test, 3) association between critical region viewing for scenes paired with *item* orienting questions during study and *item* corrected recognition scores, and 4) association between critical region viewing for scenes paired with *relational* orienting questions during study and *relational* corrected recognition scores. Additionally, Pearson’s correlations were calculated between Picture Sequence Memory Test (PSM) scores, corrected recognition scores, and viewing during test (i.e., detail-based proportion of total viewing time and first gaze duration). Two-tailed *p*-values are reported for each correlation. We used Fisher’s *r*-to-*z* transformation to statistically compare correlations between groups. Pearson’s correlation coefficients were transformed into *z*-scores using Fisher’s transformation formula: *z* = ½*ln ((1 + *r*)/(1-*r*)). *Z*-scores for each group were then statistically compared using the test statistic: *z_observed_* = (*z_1_*-*z_2_*)/sqrt ((1/(*N_1_*-3)) + (1/(*N_2_*-3)). Using a *p*-value of 0.05 to determine statistical significance, a *z_observed_* value > +1.96 or < −1.96 was considered significant.

## 3. Results

### 3.1. Behavioral performance

#### 3.1.1. Orienting questions (Study Block 2)

Two autistic participants were removed from the orienting question analysis because they used the wrong buttons to make responses on a subset of trials; therefore, analyses were based on data from 16 autistic participants and 20 non-autistic participants. Most often, participants made correct responses to the orienting questions (autistic participants: *M* = 89.32%, *SD* = 7.97%; non-autistic participants: *M* = 89.90%, *SD* = 7.41%). There was no significant difference in orienting question response accuracy between autistic and non-autistic participants, *t* (34) = 0.22, *p* = 0.83, Cohen’s *d* = 0.08, *BF_01_* = 4.01.

#### 3.1.2. Recognition

On average, scenes were most often identified correctly at test (autistic participants: *M* = 81.16% correct, *SD* = 11.54%; non-autistic participants: *M* = 87.03% correct, *SD* = 7.14%). Further evaluation of the data indicated that more than half of the scenes were recognized correctly and with high confidence by both groups (autistic participants: *M* = 51.56% correct high confidence trials, *SD* = 18.86%; non-autistic participants: *M* = 60.31% correct high confidence trials, *SD* = 18.04%). Table 3 provides a full accounting of accuracy and confidence across scene types, for both groups.

**Table 3:** Scene recognition accuracy and confidence ratings for autistic and non-autistic participants.

	Autistic (*n* = 18)						Non-autistic (*n* = 20)					
	Correct Low	Correct Mid	Correct High	Incorrect Low	IncorrectMid	Incorrect High	Correct Low	Correct Mid	Correct High	Incorrect Low	Incorrect Mid	Incorrect High
Repeat	11.46 (13.77)	37.15 (27.41)	26.39 (26.82)	5.21 (7.50)	11.81 (11.72)	7.99 (15.58)	2.81 (6.87)	48.75 (25.94)	31.56 (24.29)	3.75 (5.88)	8.44 (9.78)	4.69 (9.70)
Item	2.78 (6.15)	21.88 (28.70)	53.47 (25.92)	4.86 (6.63)	7.29 (6.52)	9.72 (15.93)	1.88 (4.58)	15.00 (11.54)	65.94 (21.12)	2.19 (4.19)	10.94 (11.63)	4.06 (6.81)
Relation	3.82 (11.77)	22.22 (24.27)	53.47 (29.80)	2.43 (3.80)	10.07 (11.37)	7.99 (13.86)	3.75 (4.71)	13.75 (14.28)	70.00 (19.72)	1.25 (4.35)	9.69 (12.08)	1.56 (3.99)
Novel	2.43 (3.80)	16.67 (18.19)	72.92 (24.06)	1.74 (2.88)	4.51 (7.05)	1.74 (3.59)	4.69 (6.69)	16.25 (20.72)	72.75 (27.70)	1.88 (2.94)	2.81 (5.16)	0.063 (1.92)
Overall	5.12 (6.96)	24.48 (19.20)	51.56 (18.86)	3.56 (2.39)	8.41 (5.95)	6.85 (11.27)	3.28 (4.39)	23.44 (13.50)	60.31 (18.04)	2.27 (2.75)	7.97 (6.08)	2.73 (4.56)

Data are reported as mean percentages (standard deviations) for test trials identified correctly or incorrectly, subdivided by confidence, and organized by group and scene type.

##### 3.1.2.1. Memory for scenes

**Figure 4:**
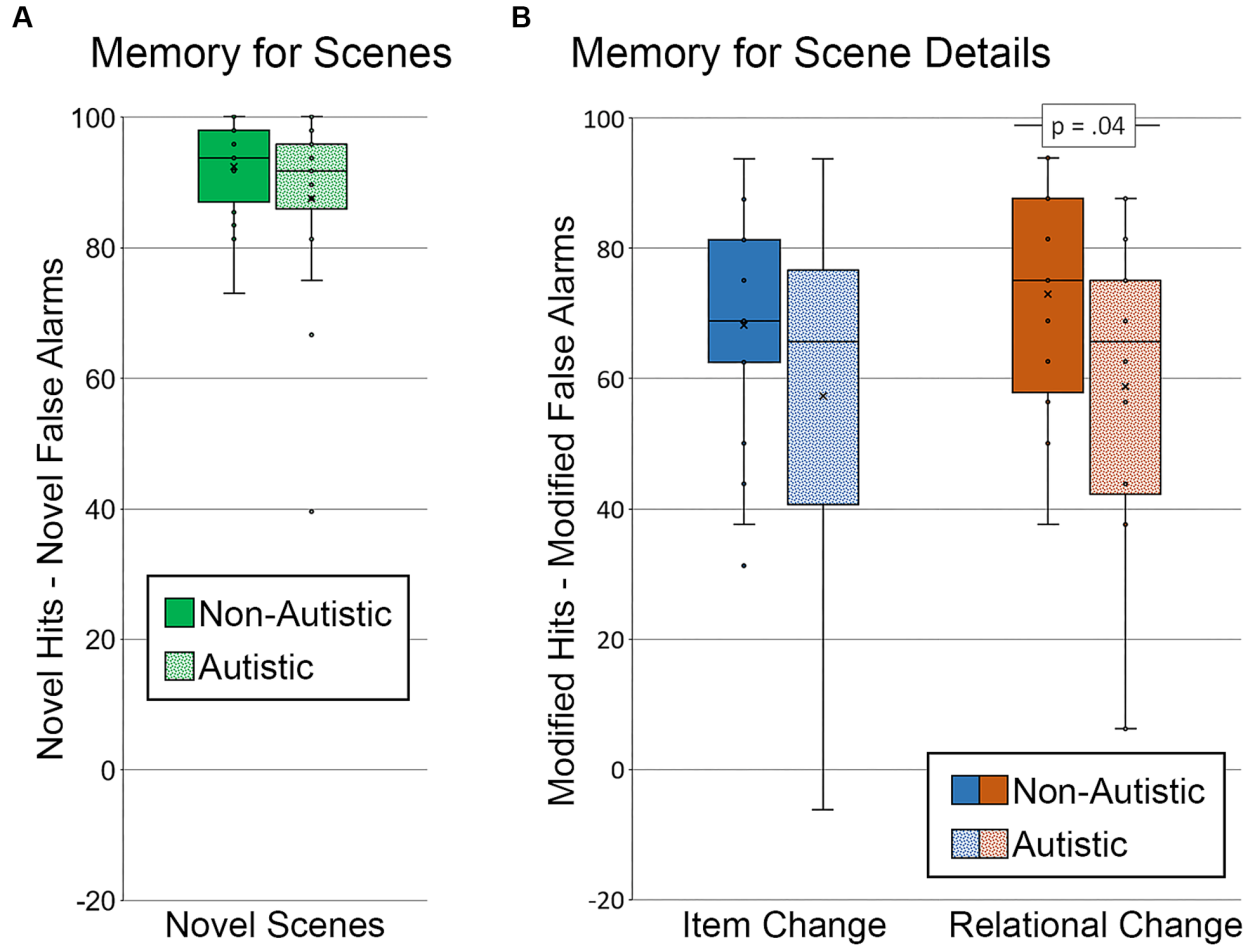
Recognition memory for scenes and scene details. Corrected recognition accuracy for **(A)** memory for scenes index (percentage of novel hits – percentage of novel false alarms) by group. Corrected recognition accuracy for **(B)** memory for scene details index (percentage of modified hits – percentage of modified false alarms) by group and scene type. Error bars represent standard error of the mean.

To determine whether there were general differences in memory for scenes, corrected recognition scores were calculated by subtracting novel false alarms (repeated and modified scenes called “new”) from novel hits for each group of participants (see Figure 4A). Results from an independent samples *t*-test indicated that there was not a significant between-groups difference in the ability to distinguish new from old scenes, *t* (36) = 1.31, *p* = 0.20, Cohen’s *d* = 0.43, *BF_01_* = 2.01.

##### 3.1.2.2. Memory for scene detail

Two corrected recognition scores sensitive to memory for detail were calculated by subtracting the percentage of modified false alarms (i.e., repeated scenes called “modified”) from the percentage of modified hits, one for scenes with item changes and one for scenes with relational changes (see Figure 4B). A repeated measures ANOVA, with factors for the group (autistic, non-autistic), scene type (item change, relational change), and their interaction, was calculated. There was a marginal effect of group, *F* (1, 36) = 3.53, *p* = 0.07, *η_p_^2^* = 0.09, but neither the main effect of scene type nor the interaction was significant, *F’*s ≤ 1.53, *p’*s ≥ 0.22, *η_p_^2^*’s ≤ 0.04. As was done by Cooper et al. (2015), independent samples *t*-tests were calculated to determine whether the group difference was significant for item changes, relational changes, or both. There was no significant group difference in corrected recognition scores sensitive to memory for item changes, *t* (36) = 1.46, *p* = 0.15, Cohen’s *d* = 0.48, *BF_01_* = 1.69. There was, however, a significant group difference in corrected recognition scores sensitive to relational memory, *t* (36) = 2.10, *p* = 0.04, Cohen’s *d* = 0.68, *BF_01_* = 0.67. This group difference in relational memory was marginal after adjusting for age and gender, *F* (1, 34) = 3.83, *p* = 0.059, *η_p_^2^* = 0.10.

##### 3.1.2.3. High confidence recognition

**Figure 5:**
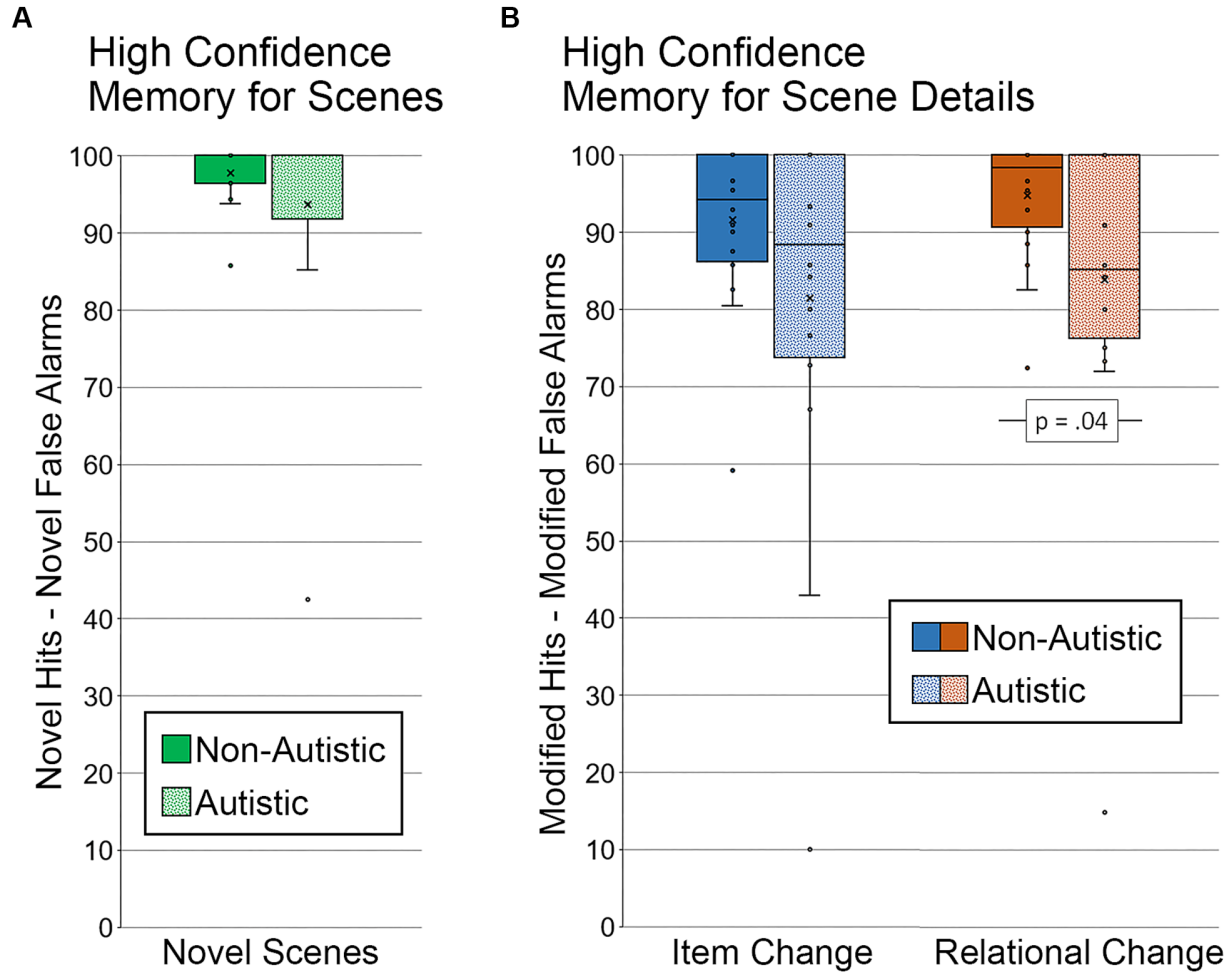
Recognition memory for scenes and scene details for high confidence correct trials. **(A)** Memory for scenes (percentage of novel hits – percentage of novel false alarms) by group, limited to scenes identified correctly and with high confidence. **(B)** Memory for scene details (percentage of modified hits – percentage of modified false alarms) by group and scene type, limited to scenes identified correctly and with high confidence. Error bars represent standard error of the mean.

Since specific weaknesses in recollection and high-confidence responding are reported in autism (e.g., Bowler et al., 2007; Cooper et al., 2015), we also examined whether group differences in memory for scenes and memory for scene detail were evident when analyses were limited to trials with high-confidence responses (see Figure 5). Two participants, one from each group, were excluded from the memory for scenes analysis because there were either no high-confidence hits for novel scenes or no high-confidence false alarms for repeated and modified scenes. Results from an independent samples *t*-test indicated that there was not a significant group difference in memory for scenes, *t* (34) = 1.20, *p* = 0.24, Cohen’s *d* = 0.40, *BF_01_* = 2.22 (see Figure 5A).

Next, we examined high-confidence memory for scene detail. Two autistic participants were excluded from this analysis. One participant did not have any high-confidence hits for scenes with an item change, and the other participant did not have any high-confidence hits for either type of manipulated scene. In addition, because several participants did not have any high-confidence false alarms to repeated scenes (i.e., repeated scenes called “modified), the false alarm rate was calculated by including novel scenes (i.e., both repeated and novel scenes called “modified” with high confidence were included in the calculated false alarm rate). Results from a repeated measures ANOVA revealed marginal effects of scene type (item change, relational change), *F* (1, 34) = 3.07, *p* = 0.09, *η_p_^2^* = 0.08, and group (autistic, non-autistic), *F* (1, 34) = 3.86, *p* = 0.06, *η_p_^2^* = 0.10, but the interaction was not significant, *F* (1, 34) = 0.08, *p* = 0.79, *η_p_^2^* = 0.002. As above, results from independent samples *t*-tests indicated that there was a significant group difference in relational memory, *t* (34) = 2.16, *p* = 0.04, Cohen’s *d* = 0.72, *BF_01_* = 0.60, but not item memory, *t* (34) = 1.67, *p* = 0.11, Cohen’s *d* = 0.56, *BF_01_* = 1.27 (see Figure 5B). In this case, the group difference for relational memory remained significant after adjusting for age and gender, *F* (1, 32) = 4.49, *p* = 0.042, *η_p_^2^* = 0.12.

### 3.2. Viewing behavior

#### 3.2.1. Study blocks

One objective of this work was to determine whether there were group differences in viewing behavior during encoding that might correspond to differences in the operation of cognitive processes that can affect memory performance (e.g., attention to critical scene regions). Two measures were used to examine between-groups differences in scene viewing during the study blocks: number of scene fixations and proportion of total viewing time directed to the filled critical region of encoded scenes.

##### 3.2.1.1. Number of fixations to studied scenes

First, we calculated the average number of fixations to whole scenes, collapsed across conditions, and without considering specific regions of interest. For Study Block 1, there was not a significant difference in the average number of scene fixations between autistic (*M* = 24.44*, SD* = 3.59) and non-autistic participants (*M* = 22.98*, SD* = 2.70), *t* (36) = 1.43, *p* = 0.16, Cohen’s *d* = 0.46, *BF_01_* = 1.75. However, for Study Block 2, autistic participants (*M* = 15.39*, SD* = 1.41) made significantly more fixations to scenes than non-autistic participants (*M* = 13.87*, SD* = 1.69), *t* (36) = 2.98, *p* = 0.005, Cohen’s *d* = 0.98, *BF_01_* = 0.12. The overall decrease (for both groups) in the number of fixations across study blocks is at least in part due to the reduction in scene presentation time (i.e., 8 s in Study Block 1 versus 5 s in Study Block 2).

##### 3.2.1.2. Proportion of total viewing time to the filled critical region of studied scenes

Next, we examined whether there were differences in the proportion of total viewing time directed to the *filled* critical region of studied scenes – the location occupied by an object. We did not examine the proportion of total viewing time to empty critical regions because they were not meaningful (i.e., had never been occupied by an object) at this point in the experiment. For this analysis, all of the studied scenes, regardless of whether they went on to be repeated or manipulated during test, were subdivided by the type of orienting question (item-specific, relational) they were paired with during Study Block 2. Repeated measures ANOVAs with the factors group (autistic, non-autistic) and question type (item-specific, relational) were calculated separately for Study Block 1 and Study Block 2.

**Figure 6:**
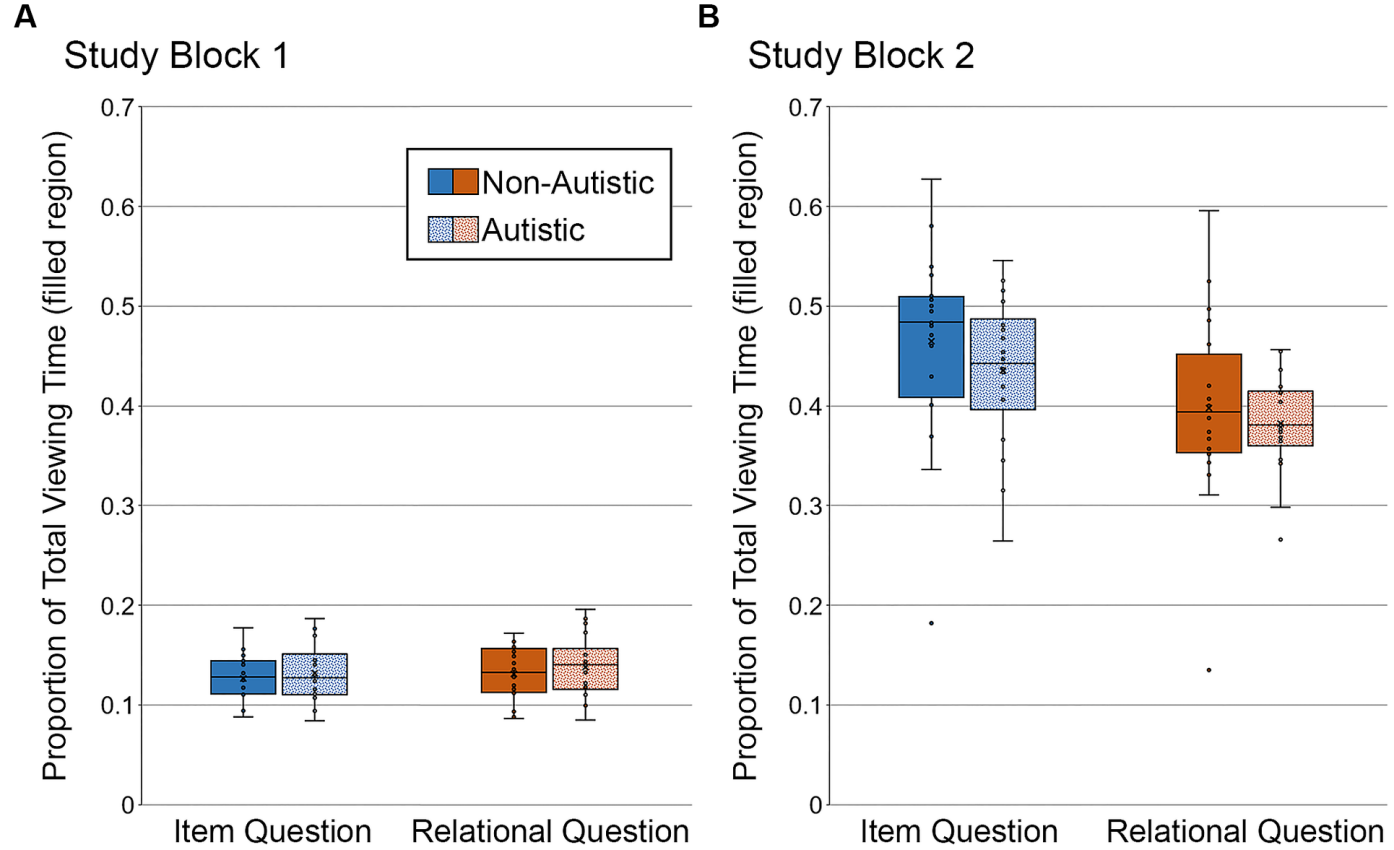
Proportion of total viewing time directed to the filled critical region of to-be-manipulated scenes in **(A)** Study Block 1 and **(B)** Study Block 2, subdivided by group and question type. Error bars represent standard error of the mean.

In Study Block 1 (see Figure 6A), there were no significant main effects or interactions, *F*’s ≤ 1.15, *p*’s ≥ 0.29, *η_p_^2^*’s ≤ 0.031. Bayes factors were in favor of the null hypothesis – i.e., no group differences in the proportion of total viewing time directed to the filled region of scenes paired with item-specific or relational questions, *BF_01_* = 3.65 and *BF_01_* = 3.23, respectively.

In Study Block 2 (see Figure 6B), there was a significant main effect of question type, *F* (1, 36) = 41.56, *p* < 0.001, *η_p_^2^* = 0.54, with more viewing directed to the filled region for scenes paired with item-specific than relational questions. There was no significant main effect of group and no significant interaction, *F*’s ≤ 0.80, *p*’s ≥ 0.38, *η_p_^2^*’s ≤ 0.022. Bayes factors indicated that the data were inconclusive regarding group differences in viewing directed to the filled region of scenes paired with item-specific questions, *BF_01_* = 2.66, but were in favor of the null hypothesis for scenes paired with relational questions, *BF_01_* = 3.54.

As can be seen in Figure 6, the proportion of total viewing time directed to the filled critical region was greater in Study Block 2 than in Study Block 1. This is because orienting questions, used in Study Block 2, required participants to inspect the critical objects and/or their relative locations. Reduced viewing of the filled critical region for scenes paired with relational (versus item-specific) orienting questions in Study Block 2 likely occurs because these questions encouraged exploration of an object relative to something else in the scene. In contrast, item-specific orienting questions asked about characteristics of the object itself.

##### 3.2.1.3. High confidence proportion of total viewing time to the filled critical region

To determine whether there were any between-groups differences in viewing directed to the filled critical region of studied scenes that went on to be correctly recognized and endorsed with high confidence, we backsorted the study phase data by test block performance. In other words, we binned study trials by subsequent test phase accuracy (i.e., correct, incorrect) and recognition confidence (i.e., high, middle, low). This analysis was limited to scenes that would go on to be modified in the test block. Repeated scenes were excluded because several participants did not have any high-confidence correct recognition responses in the repeated scene condition. In addition, two participants from the autistic group were excluded from these backsorted analyses. In one case, there were no high-confidence correct responses to scenes with item changes; in the other case, there were no high-confidence correct responses to any of the manipulated scenes. Repeated measures ANOVAs with the factors scene type (item, relational) and group (autistic, non-autistic) were calculated separately for Study Block 1 and Study Block 2.

**Figure 7:**
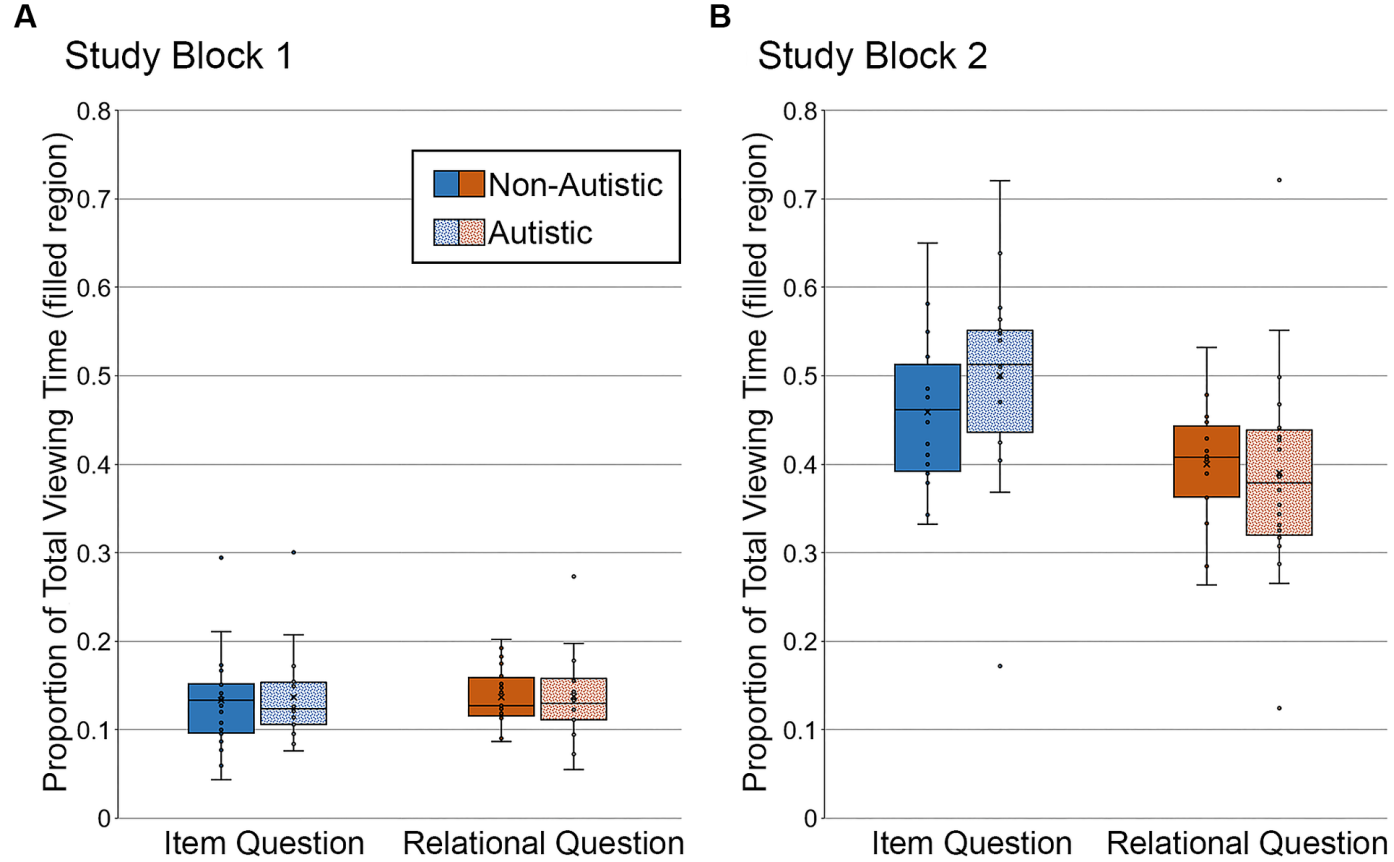
Proportion of total viewing time directed to the filled critical region of to-be-manipulated scenes in **(A)** Study Block 1 and **(B)** Study Block 2, subdivided by group and question type, and limited to the subset of scenes identified correctly and with high confidence. Error bars represent standard error of the mean.

In Study Block 1 (see Figure 7A), there was no difference in the proportion of total viewing time directed to the filled region across question types or groups, *F*’s ≤ 0.06, *p*’s ≥ 0.81, *η_p_^2^*’s ≤ 0.002, nor was there a significant interaction, *F* (1, 34) = 0.009, *p* = 0.92, *η_p_^2^* < 0.0001. As when analyses were based on all trials, Bayes factors were in favor of the null hypothesis – i.e., no group differences in the proportion of total viewing time directed to the filled region of scenes accompanied by item-specific or relational questions, *BF_01_* = 4.02 and *BF_01_* = 4.08, respectively.

In Study Block 2 (see Figure 7B), there was a significant effect of question type, *F* (1, 34) = 26.05, *p* < 0.001, *η_p_^2^* = 0.43, but there was not a significant group effect or a question type by group interaction, *F*’s ≤ 2.45, *p*’s ≥ 0.13, *η_p_^2^*’s ≤ 0.067. As when analyses were based on all trials, Bayes factors indicated that the data were inconclusive regarding group differences in viewing directed to the filled region of scenes associated with item-specific questions, *BF_01_* = 2.16, but were in favor of the null hypothesis for scenes paired with relational questions, *BF_01_* = 3.94.

Once again, more time was spent looking at the filled region of scenes paired with item-specific questions than with relational questions, likely due to differences in processing requirements associated with these types of questions (see Figure 7).

#### 3.2.2. Test block

Another major objective of this work was to assess group differences in viewing behavior during the test phase. As described above, we calculated two difference scores – *memory for repetition* (repeated scene viewing minus novel scene viewing) and *memory for detail* (modified scene viewing minus repeated scene viewing) – to examine viewing patterns during test. Difference scores were calculated for two eye-movement measures: proportion of total viewing time directed to critical scene region(s) and the duration of the first gaze (in ms) made to the filled region. Independent samples *t*-tests were calculated to compare the proportion of total viewing time directed to the filled critical region in item scenes, the filled critical region in relational scenes, and the empty critical region in relational scenes for the proportion of viewing time measure. First gaze analyses were limited to the filled region.

##### 3.2.2.1. Proportion of viewing time to filled and empty critical regions of test scenes

**Figure 8:**
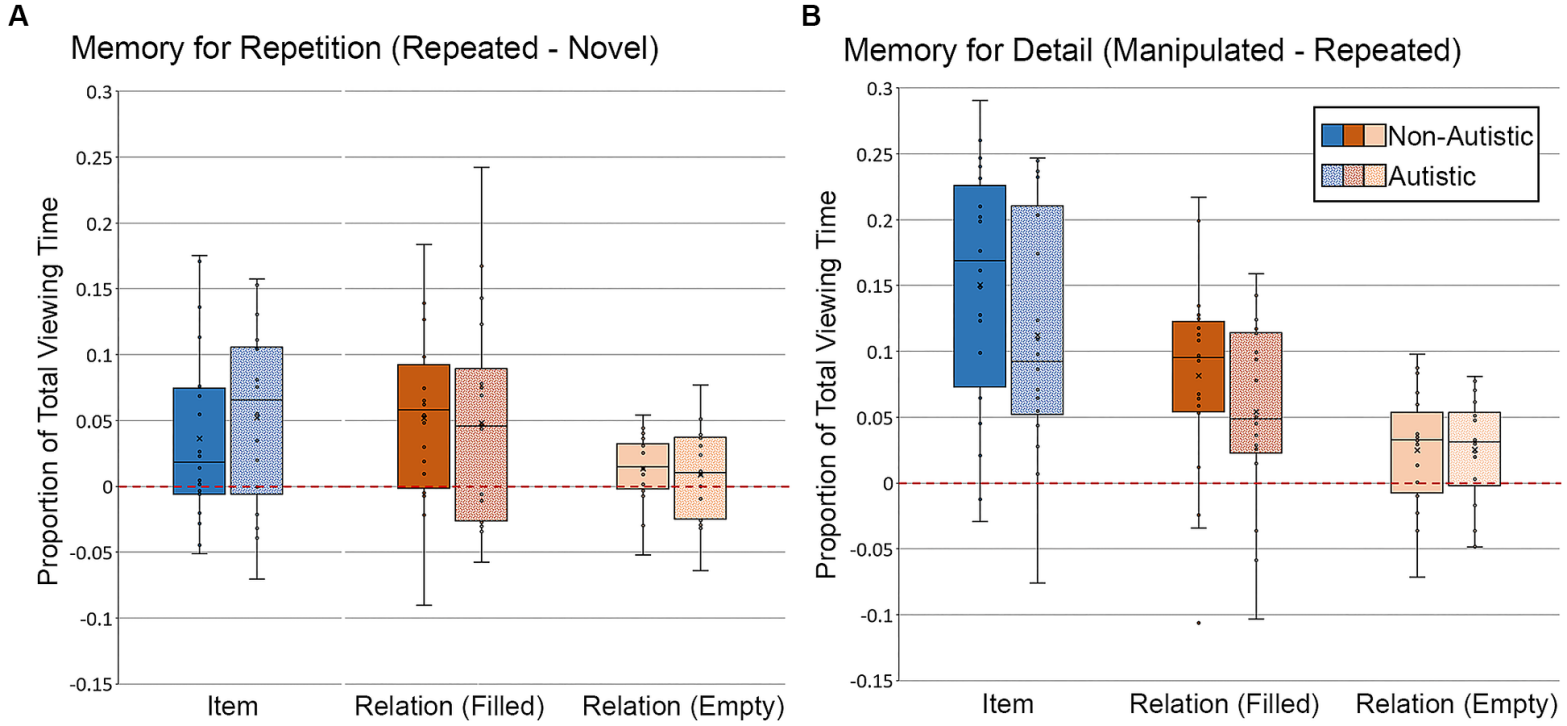
Proportion of total viewing time directed to critical regions of scenes for **(A)** Memory for Repetition index (viewing to repeated scenes – viewing to novel scenes) and **(B)** Memory for Detail index (viewing to manipulated scenes – viewing to repeated scenes), subdivided by group, scene type, and critical region for scenes presented during Test Block. Error bars represent standard error of the mean.

First, we examined differences in the proportion of total viewing time to critical regions of test scenes. For scenes in the item condition, there were no significant group differences in repetition- or detail-based proportion of total viewing time to the filled critical region, *t*’s ≤ 1.26, *p*’s ≥ 0.22, Cohen’s *d*’s ≤ 0.43, *BF_01_* = 3.36 and 2.13, respectively. Likewise, for scenes in the relational condition, there were no significant group differences in repetition- or detail-based proportion of total viewing time to either the filled or empty critical region, *t*’s ≤ 1.14, *p*’s ≥ 0.26, Cohen’s *d*’s ≤ 0.38, *BF_01_* repetition filled = 4.17, *BF_01_* repetition empty = 3.82, *BF_01_* detail filled = 2.40, *BF_01_* detail empty = 4.21. As can be seen in Figure 8A, participants from both groups spent more time looking at the critical region(s) of repeated scenes than the same region(s) of novel scenes (i.e., positive-going difference scores), a likely consequence of the orienting questions during encoding. Participants from both groups also spent more time looking at the critical region(s) of manipulated scenes than the same region(s) of repeated scenes (i.e., positive-going difference scores), an index of memory for scene detail, as illustrated in Figure 8B.

###### 3.2.2.1.1. High confidence proportion of total viewing time to filled and empty regions

**Figure 9:**
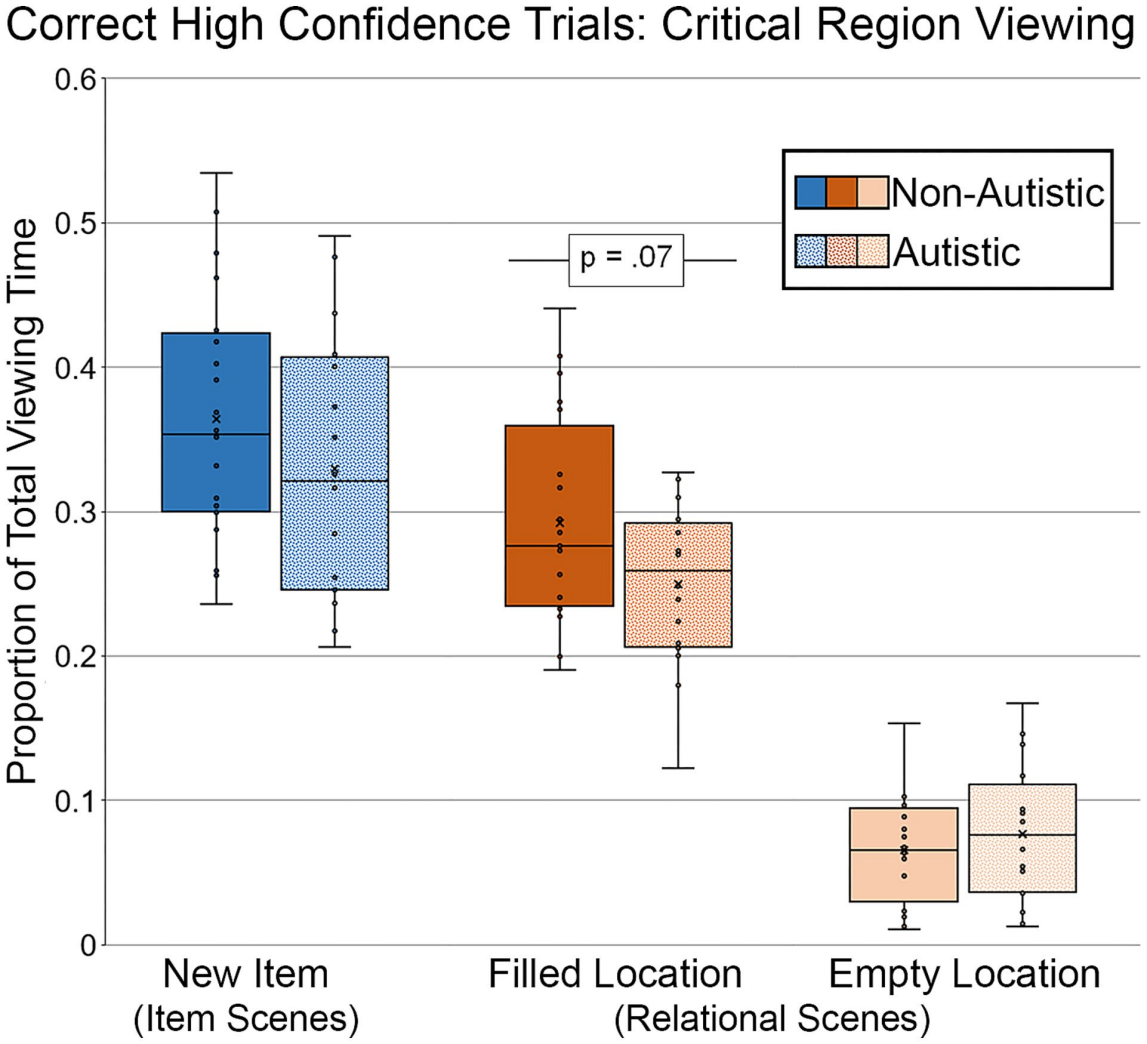
Proportion of total viewing time directed to critical regions of scenes, subdivided by group, scene type, and critical region for scenes presented during Test Block, and limited to scenes that were identified correctly with high confidence. Error bars represent standard error of the mean.

Targeted analyses were performed to examine whether there were any viewing time differences for scenes correctly recognized and endorsed with high confidence (see Figure 9). For this analysis, like before, data from 16 autistic participants and 20 non-autistic participants were included. As above, two autistic participants were dropped from the analysis because there were no high-confidence correct trials for modified scenes with item or item and relational manipulations. Furthermore, difference scores (i.e., memory for repetition and detail) were not calculated for the high-confidence analyses because several participants from both groups identified fewer than 3 repeated scenes correctly with high confidence. Therefore, these analyses were based on proportion of total viewing time to the critical region(s) of modified scenes recognized correctly with high-confidence responses. Results from an independent-samples *t*-test indicated that there was not a significant group difference in the proportion of total viewing time directed to the filled region of scenes with an item change, *t* (34) = 1.15, *p* = 0.26, Cohen’s *d* = 0.33, *BF_01_* = 2.32, but that this difference was marginal for the filled region of scenes with a relational change, *t* (34) = 1.90, *p* = 0.066, Cohen’s *d* = 0.61, *BF_01_* = 0.91. The proportion of total viewing time was lower for the autistic group than for the non-autistic group. There was no significant group difference in proportion of viewing to the empty critical region, *t* (34) = 0.77, *p* = 0.45, Cohen’s *d* = 0.25, *BF_01_* = 3.17. The group difference in the relational condition for the filled region became significant after adjusting for age and gender, *F* (1, 32) = 4.21, *p* = 0.048, *η_p_^2^* = 0.12.

##### 3.2.2.2. First gaze duration to the filled region of test scenes

**Figure 10:**
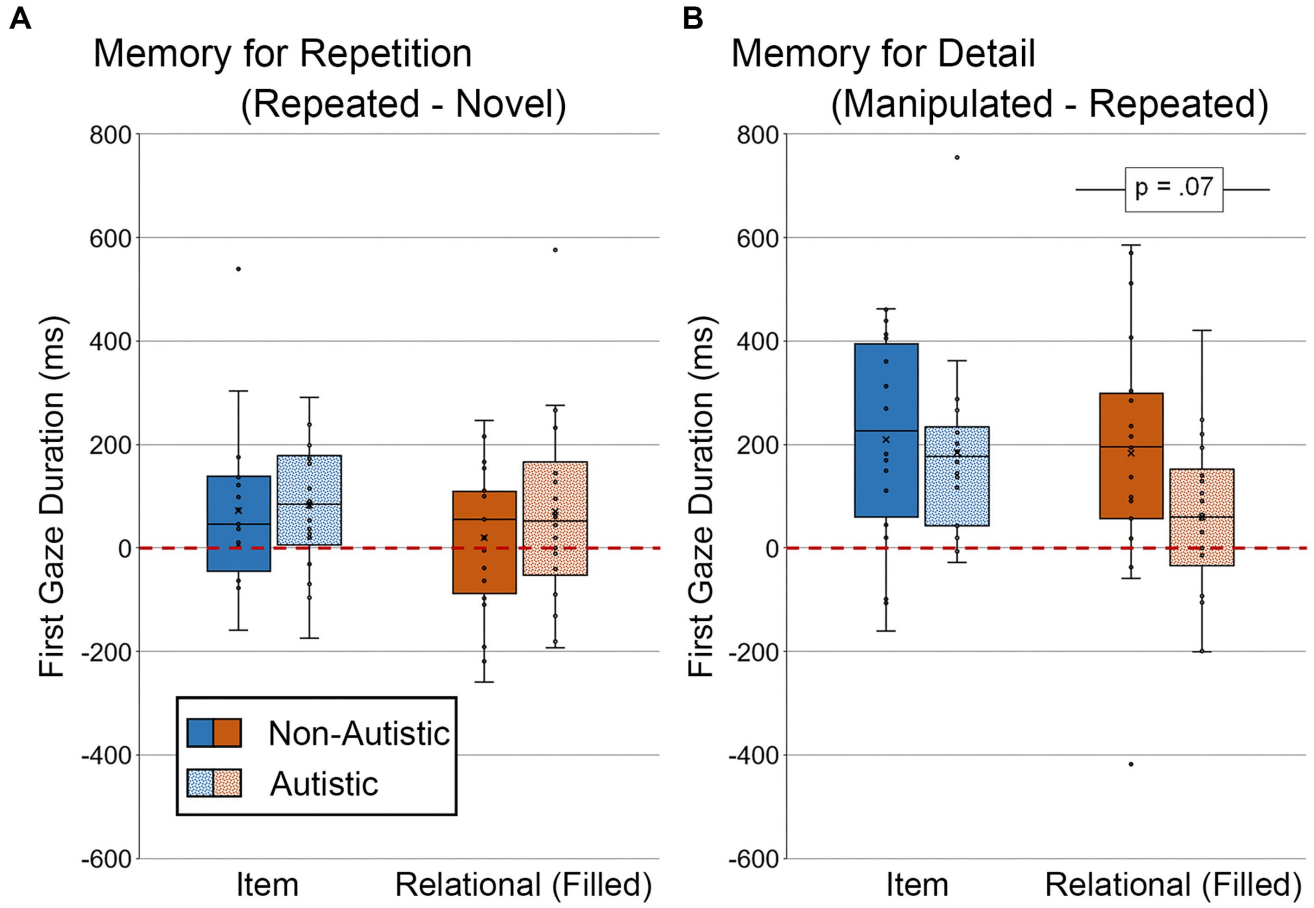
First gaze duration (ms) to filled critical region of scenes for **(A)** Memory for Repetition index (viewing to repeated scenes – viewing to novel scenes) and **(B)** Memory for Detail index (viewing to manipulated scenes – viewing to repeated scenes), subdivided by group and scene type for scenes presented during Test Block. Error bars represent standard error of the mean.

We also examined differences in the duration of the first gaze made to the filled critical region (see Figure 10). For scenes in the item condition, there were no significant group differences in the duration of the first gaze directed to the filled critical region for the repetition-based difference score or the detail-based difference score, *t*’s ≤ 0.41, *p*’s ≥ 0.69, Cohen’s *d*’s ≤ 0.13, *BF_01_*’s ≥ 3.91. For scenes in the relational condition, there was no significant group difference in first gaze for the repetition-based difference score, *t* (36) = 0.93, *p* = 0.72, Cohen’s *d* = 0.30, *BF_01_* = 2.88, but there was a marginal group difference in duration of first gaze for the detail-based difference score, *t* (36) = 1.88, *p* = 0.068, Cohen’s *d* = 0.62, *BF_01_* = 0.94, with non-autistic participants spending more time looking at the now-filled regions relative to autistic participants. This group difference for detail-based viewing in the relational condition remained marginally significant after adjusting for age and gender, *F* (1, 34) = 2.96, *p* = 0.095, *η_p_^2^* = 0.080.

###### 3.2.2.2.1. High confidence first gaze duration to the filled region

**Figure 11:**
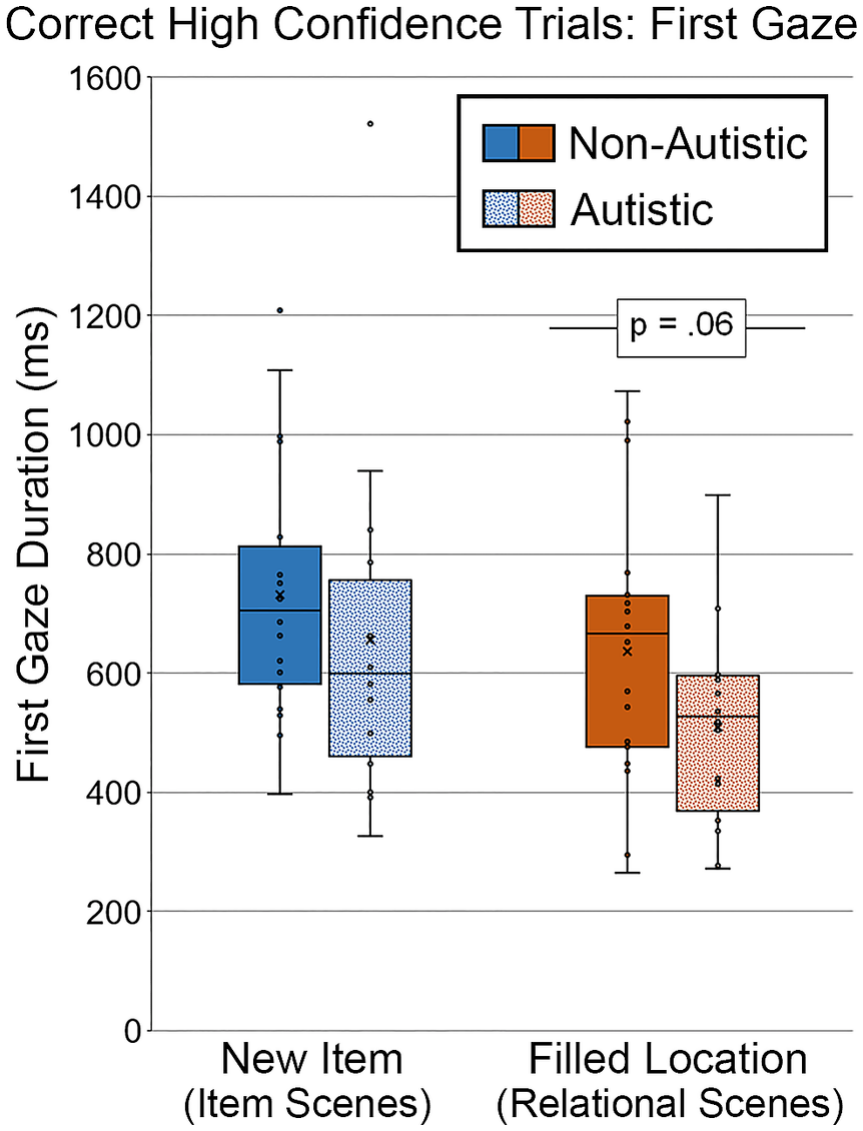
First gaze duration (ms) to the filled critical region of scenes, subdivided by group and scene type during Test Block, and limited to scenes identified correctly and with high confidence. Error bars represent standard error of the mean.

Analyses were performed to determine whether there were differences in first gaze duration toward scenes that were identified correctly with high confidence (see Figure 11). As above, two autistic participants were dropped from this analysis, and difference scores were not calculated because there were so few high-confidence trials for repeated scenes. For scenes with an item change, there was no significant group difference in duration of first gaze, *t* (34) = 0.92, *p* = 0.36, Cohen’s *d* = 0.30, *BF_01_* = 2.84. However, there remained a marginal group difference in first gaze to the filled region for scenes with relational changes, *t* (34) = 1.91, *p* = 0.065, Cohen’s *d* = 0.65, *BF_01_* = 0.89. This group difference in the relational condition remained marginal after adjusting for age and gender, *F* (1, 32) = 3.03, *p* = 0.091, *η_p_^2^* = 0.087.

### 3.3. Correlation analyses

Pearson’s correlations (*r*) were calculated to determine whether viewing time to the filled critical region during the study blocks was associated with memory-based (i.e., detail-based) viewing effects and/or recognition performance in the test block. For this set of analyses the average proportion of total viewing time to the filled region of studied scenes was calculated separately for scenes paired with item-specific and relational orienting questions, collapsed across study blocks, for each participant. This grand average (i.e., proportion of total viewing time directed to the filled region during the study phase) was used in all reported analyses. Two test block measures were used to determine whether study phase viewing time was correlated with test block viewing directed to the *filled* critical region for item-specific and relational scenes separately. These two measures were the *memory for detail* difference scores for 1) proportion of viewing time and 2) first gaze duration, which provide us an estimate of viewing time to the critical region due to memory for the original item or the spatial position of the critical item in the test block. Corrected recognition scores for scenes with an item and relational change were also included in the correlation analyses.

#### 3.3.1. Correlations between study and test viewing

First, we compared study and test viewing patterns. For scenes containing an item change, there were no significant correlations between study and test phase viewing patterns for either autistic participants, *r*’s ≤ 0.34, *p*’s ≥ 0.17, or non-autistic participants, *r*’s ≤ 0.16, *p*’s ≥ 0.50. Additionally, there were no significant correlations between study and test phase viewing for scenes containing a relational change for autistic participants, *r*’s ≤ 0.25, *p*’s ≥ 0.32, or non-autistic participants, *r*’s ≤ 0.24, *p*’s ≥ 0.30. Unsurprisingly, there were no significant group differences in correlations between study and testing viewing patterns, *z*’s ≤ 0.54, *p*’s > 0.05.

#### 3.3.2. Correlations between study viewing and recognition performance

Next, we calculated correlations between study viewing patterns and test recognition memory. For scenes that underwent an item change, there was a significant positive correlation between study viewing and item memory for non-autistic participants, *r* = 0.51, *p* = 0.022, but no significant correlation for autistic participants, *r* = −0.20, *p* = 0.43. In contrast, for scenes that underwent a relational change, there was a marginal negative correlation between study viewing and relational memory for autistic participants, *r* = −0.45, *p* = 0.059, but no significant correlation for non-autistic participants, *r* = 0.19, *p* = 0.43. However, only the correlation between study viewing and item recognition memory was significantly different between groups, *z* = −2.16, *p* < 0.05. All other between-group differences in these correlations were not significant, *z*’s ≤ 1.91, *p*’s > 0.05.

#### 3.3.3. Correlations between PSM scores, test viewing, and recognition performance

Finally, we calculated Pearson’s correlations to compare Picture Sequence Memory Test (PSM) scores with item-specific and relational corrected recognition memory scores and detail-based (i.e., memory-based) viewing patterns during the Test Block. There was a significant positive correlation between PSM scores and item memory for non-autistic participants, *r* = 0.44, *p* = 0.051, but not for autistic participants, *r* = −0.16, *p* = 0.53. In contrast, there was a significant negative correlation between PSM scores and first gaze duration for scenes in the relational condition for autistic participants, *r* = −0.53, *p* = 0.025, but not for non-autistic participants, *r* = −0.11, *p* = 0.64. None of the between-group differences in these correlations were significant, *z*’s ≤ 1.79, *p*’s > 0.05.

## 4. Discussion

The current study examined whether memory-specific viewing patterns to realistic, non-social scenes differed between autistic and non-autistic individuals. Here, we employed an eye-tracking paradigm that equated difficulty across item-specific and relational conditions (i.e., Hannula et al., 2010b; Cooper et al., 2015) to control for potential differences in task complexity that may have contributed to past findings. In addition, we used both direct (i.e., explicit responses) and indirect (i.e., eye movements) measures of memory to examine performance. Orienting question accuracy was not significantly different between groups during Study Block 2, suggesting that both groups attended to relevant scene regions when prompted. In Study Block 2, autistic individuals made more scene fixations than non-autistic participants, but there was no evidence for differential viewing of the filled critical region across groups in either study block. Therefore, this difference in total number of scene fixations did not affect time spent viewing the scene region that would be modified in the item and relational conditions during test.

Behaviorally, both autistic and non-autistic participants could distinguish between studied and non-studied scenes. While there was no significant difference in accuracy for scenes that underwent an item change, autistic participants showed a marginal reduction in relational memory accuracy across all trials and a significant reduction in relational memory accuracy for high-confidence trials relative to their non-autistic peers. Additionally, evaluation of the eye-tracking data indicated that both groups showed evidence of memory-based viewing effects (i.e., greater viewing of filled regions of modified scenes relative to analogous regions of yoked novel and repeated scenes) during test. However, autistic individuals spent a marginally smaller proportion of total viewing time on, and demonstrated marginally shorter initial gazes toward, relational changes in scenes relative to their non-autistic counterparts for all trials (for the gaze duration index) and for high-confidence trials (for both the proportion of total viewing time and gaze duration indices). Further, the group difference in proportion of total viewing time for high-confidence trials was significant when adjustments were made for age and gender. Taken together, our recognition and eye-movement measures provide converging evidence for a selective weakness in relational memory in autism.

Correlational analyses revealed no significant between-group differences in associations between performance on a standardized episodic memory task (i.e., Picture Sequence Memory Test) and viewing during test or recognition memory. However, viewing patterns during the study phase were correlated with subsequent recognition accuracy, as has been reported previously (Loth et al., 2011; Ring et al., 2017; Cooper et al., 2017a). Specifically, for scenes assigned to the item condition, there was a positive association between critical region viewing during the study phase and the successful recognition of scenes with item-specific changes for the non-autistic group, but no similar effect for the autistic group, and this between-groups difference was statistically significant. In contrast, there was a marginal, negative association between study phase viewing and relational memory for the autistic group, though here, there was not a significant between-groups difference. Overall, these outcomes suggest that viewing patterns during encoding may not always predict test phase outcomes in the same way and/or to the same degree in autistic and non-autistic participants, as reported previously by Cooper et al. (2017a).

As outlined above, past work demonstrates that episodic memory processes are atypical in autism. However, the type of representational content impacted by episodic memory difficulties is contested, with some authors reporting weaknesses restricted to item-specific memory (Solomon et al., 2016; Cooper et al., 2017a) and others reporting selective relational memory difficulties (Lind and Bowler, 2009; Bigham et al., 2010; Bowler et al., 2014; Cooper et al., 2017b; Desaunay et al., 2020b), weaknesses in both item and relational memory (Massand and Bowler, 2015; Ring et al., 2016; Semino et al., 2018; Mogensen et al., 2020), or no item-specific or relational memory difficulties (Souchay et al., 2013; Lind et al., 2014; Ring et al., 2015, 2017; Hogeveen et al., 2020). One proposed explanation for contradictory findings is the differential complexity of past item-specific and relational memory tasks (see Cooper and Simons (2019) for review), an issue that Cooper et al. (2015) attempted to address by utilizing a behavioral task that ours is similar to, with materials developed to equate item-specific and relational memory processing demands. Their work showed that autistic individuals identified fewer scenes with item and relational changes than their non-autistic peers, a finding taken as evidence for a potential weakness in both item-specific and relational memory (Cooper et al., 2015).

Because task demands of the current study were closely matched to Cooper et al. (2015), one may question why we only observed group differences in relational recognition performances rather than in both item-specific and relational recognition performances. Importantly, it should be noted that the sample size of the current study was sufficient to detect large effect sizes (*d* = 0.9) but may have been underpowered to detect more subtle effects. However, we did observe significant and marginal group differences in relational memory and memory-based viewing effects for scenes with a relational change. Further, Cooper et al. (2015) reported larger effect sizes for their item memory group differences as compared to their relational memory group differences. Thus, our sample size should have been sufficient to detect a group difference in both item and relational memory. It is possible that the addition of a second study block, which provided participants with a directed viewing task (i.e., via orienting questions) as well as a second opportunity to view scenes, mitigated attentional or executive processing difficulties that would have otherwise impacted explicit recognition memory for items in the autistic group in our study. Indeed, this hypothesis aligns with past work demonstrating improvements in recognition memory performance in autistic participants when explicit encoding instructions are provided (Gaigg et al., 2008; Bowler et al., 2010; Cooper et al., 2017a) and is consistent with the *task support hypothesis*, which proposes that autistic individuals’ memory improves when they are provided with “supports” during a memory task (e.g., cues in a recognition memory paradigm; Bowler et al., 2004). However, despite the use of a controlled encoding task here, relational memory could not be rescued in the autistic group relative to the non-autistic group, an outcome consistent with past findings that suggest relational memory is selectively or disproportionately compromised in autistic individuals (Lind and Bowler, 2009; Bigham et al., 2010; Bowler et al., 2014; Cooper et al., 2017b; Desaunay et al., 2020b). The group difference in relational recognition accuracy was marginal when all of the trials were included in our analyses and significant for high-confidence trials, which reinforces prior reports that autistic persons show attenuated memory confidence for correct memories (Wojcik et al., 2013; Grainger et al., 2014; Cooper et al., 2016). Importantly, significant group differences in memory confidence judgments were limited to measures that were sensitive to relational memory in the current study, a finding similar to past work that has documented reduced high-confidence, recollection-related memory in autism (Bowler et al., 2007; Cooper et al., 2015, 2017a).

A strength of the current experiment was the use of eye-tracking methods during both study and test blocks. In contrast to discrete recognition responses, eye-tracking data is recorded continuously, allowing us to examine how scenes are viewed during encoding and retrieval. Of the few previous eye-tracking studies examining encoding-related viewing behavior, none reported differences between autistic and non-autistic groups (Loth et al., 2011; Cooper et al., 2017a). This result was generally replicated here, as there was not a significant group difference in proportion of total viewing time directed to the critical object in either study block. One possibility is that well-matched viewing patterns during the study phase means that the scenes were processed comparably by participants from both groups. However, it is also possible that, while viewing patterns are similar, the depth of processing between groups, in the absence of specific task instructions, is not. The orienting questions in our experiment may have been instrumental in this regard, encouraging participants to pay close attention to the very same details of scenes that might be modified at test. Future studies, with larger sample sizes, should systematically manipulate the use of orienting questions to further examine whether and how they affect recognition performance and eye-movement-based memory effects in autism.

In contrast to results from encoding, subtle differences in retrieval-related eye movements were observed for autistic participants in the present study, in a manner that was consistent with the relational memory weakness observed in recognition memory accuracy. Past eye-tracking studies documented differences in memory-based eye-movement behaviors (Ring et al., 2017; Cooper et al., 2017a). Specifically, in a relational memory paradigm, it was reported that autistic participants spent less time viewing critical scene regions as compared to their non-autistic counterparts (Ring et al., 2017). While memory-based viewing results in our experiment trended in the same general direction, with autistic participants showing reduced viewing to critical regions associated with a relational change, our group differences were relatively small and were sometimes only observed when we analyzed high-confidence responses separately. Several factors may account for the difference in the strength of this effect between our current work and previous findings. One possibility is that our results did not reach statistical significance due to low statistical power (e.g., Bayes factors that indicated evidence for group differences was inconclusive). However, another possibility is that differences in the demands of the retrieval tasks in previously published studies and our current study affected the outcomes. For instance, participants were required to switch between two different retrieval tasks in Ring et al.’s (2017) study. Sometimes, they had to place a presented object in the location where it had been studied in the scene previously (on “explicit” trials), and sometimes they had to avoid that location, placing the object in a new spot (on “implicit” trials). This kind of task-switching may have placed greater demands on other cognitive functions, such as cognitive flexibility (i.e., set shifting), which seems to be a weakness for autistic individuals (e.g., Van Eylen et al., 2011; Andreou et al., 2022; although see Geurts et al., 2009). Importantly, results from our study suggest that eye-movement-based relational memory effects are modestly impaired even in the absence of task-switching demands and even when the encoding task encourages processing of the very same relationships that are changed during the test phase. Collectively then, these results provide converging evidence for a selective reduction in viewing effects that are sensitive to relational memory in autism.

Consistent with previous results showing differences in high confidence responding or recollection (Bowler et al., 2007; Cooper et al., 2015, 2017a) as well as with explicit recognition results reported here, when high confidence recognition trials were examined separately, marginal group differences remained and/or emerged in relational, memory-based viewing at test. These viewing time differences suggest that even when relational scenes are identified correctly with high confidence at test, there may be differences in how relationships amongst scene elements are processed by autistic individuals. Specifically, autistic individuals demonstrated a reduction in proportion of total viewing time directed to the filled region of scenes that contained a relational change and also showed shorter initial gaze durations toward critical regions of those scenes. Together with significant reductions in recognition performance for this same set of relational scenes, our results support the hypothesis that there is a disruption in recollection-related retrieval processes in autism, which appear to be selective to relational memory (Cooper et al., 2017a). Therefore, subtle differences in retrieval-related relational memory processes and/or the quality of relational memory representations (e.g., subjective quality) may exist, consistent with findings reported in past work (Lind and Bowler, 2009; Bigham et al., 2010; Bowler et al., 2014; Cooper et al., 2017b; Desaunay et al., 2020b). Of note, other processing differences, such as group differences in criteria for confidence judgments or group differences in mnemonic cues used to make confidence judgments, could partially explain the marginal effects that emerged during analysis of high-confidence trials. However, these explanations are unable to fully account for intact effects in the item-specific condition and results from the relational condition based on the full set of trials, which also provided evidence for a relational memory weakness for autistic participants.

Importantly, the absence of group differences in item-specific memory in our work should not be taken as evidence for equivalent memory processes in autistic and non-autistic individuals. For example, despite explicit memory performances that appear comparable between autistic and non-autistic individuals, electrophysiological studies report differences in magnitude and/or spatial location of event-related potentials (ERPs) associated with memory retrieval (Massand et al., 2013; Massand and Bowler, 2015; Desaunay et al., 2020b) and imaging studies document hyper-recruitment and connectivity differences between autistic and non-autistic individuals (Hogeveen et al., 2020), suggesting that compensatory neural processes may contribute to seemingly intact behavioral memory performances. Indeed, the results of correlation analyses in the present study were suggestive of processing differences between groups. Consistent with prior work (e.g., Cooper et al., 2017a), we observed a relationship between viewing during study and subsequent recognition performances. However, these relationships were different between the autistic and non-autistic groups. For example, the correlation between viewing during study and item recognition in non-autistic individuals was absent for the autistic group. Further, though the association was not significantly different between groups, the direction of a marginally significant association between viewing during study and relational memory for the autistic group was opposite that which we might expect, with a smaller proportion of viewing toward the critical region during study being associated with better relational memory in the autistic group. Altogether, these findings suggest that correlations between indirect and direct measures of memory may be sensitive to subtle differences between groups that are not observed when these types of measures are examined separately.

Several limitations of the current study should be considered. First, specific characteristics of the sample included here may have impacted our findings. For example, the autistic individuals who participated in this study were without co-morbid intellectual disability diagnoses (IQ ≥ 70); thus, results may not be generalizable to an autistic population with intellectual disability. Further, the age range of participants, spanning from adolescence to young adulthood in both groups, may have obscured or attenuated episodic memory differences between groups. Notably, the neural circuits associated with memory continue to develop from early childhood and adolescence to adulthood (Naveh-Benjamin, 2000; Grady et al., 2003; DeMaster et al., 2014). Therefore, it is possible that item memory weaknesses, for example, may only emerge later in adulthood for autistic individuals, when development of these networks is more fully matured. With these caveats in mind, the current study contributes to the growing body of evidence that documents disproportionate relational memory difficulties in autism, even when structured encoding conditions are provided and the complexity of memory tasks is equated. In future work, indirect measures of memory (i.e., eye movements) and judgments of mnemonic accuracy should be simultaneously collected because more subtle group differences may emerge when limiting analyses to high-confidence responses.

In conclusion, relational memory differences between autistic and non-autistic individuals persist, even with a controlled encoding task, and direct and indirect memory indices are useful in fully characterizing these nuanced memory effects. Reductions in recognition accuracy and memory-based viewing in the autistic group, for high confidence and correctly identified relational scenes in particular, suggest that previously reported relational memory weaknesses may have been accurately identified in past work, consistent with the *relational binding account* of episodic memory in autism (Bowler et al., 2011). Further, differences in the association between study phase viewing and recognition accuracy between groups suggest dissimilarities in underlying processes that contribute to learning and/or retrieval of learned information for autistic and non-autistic individuals. Taken together, our findings suggest differences in the integrity of relational memory representations and/or the relationships between memory subcomponents in autism.

## Data availability statement

The raw data supporting the conclusions of this article will be made available by the authors, without undue reservation.

## Ethics statement

The studies involving humans were approved by UC Davis Institutional Review Board. The studies were conducted in accordance with the local legislation and institutional requirements. Written informed consent for participation in this study was provided by the participants’ legal guardians/next of kin.

## Author contributions

GM: methodology, software, formal analysis, visualization, writing – original draft, and writing – review and editing. DH: methodology, software, formal analysis, visualization, writing – original draft, writing – review and editing, and supervision. AG: investigation and writing – review and editing. A-MI: formal analysis and writing – review and editing. JR: writing – review and editing. MS: funding acquisition (NIMH #1R01MH106518), project administration, writing – review and editing, and supervision. All authors contributed to the article and approved the submitted version.

## Conflict of interest

The authors declare that the research was conducted in the absence of any commercial or financial relationships that could be construed as a potential conflict of interest.

## Publisher’s note

All claims expressed in this article are solely those of the authors and do not necessarily represent those of their affiliated organizations, or those of the publisher, the editors and the reviewers. Any product that may be evaluated in this article, or claim that may be made by its manufacturer, is not guaranteed or endorsed by the publisher.
